# Persistent socioeconomic inequalities in cardiovascular risk factors in England over 1994-2008: A time-trend analysis of repeated cross-sectional data

**DOI:** 10.1186/1471-2458-12-129

**Published:** 2012-02-14

**Authors:** Shaun Scholes, Madhavi Bajekal, Hande Love, Nathaniel Hawkins, Rosalind Raine, Martin O'Flaherty, Simon Capewell

**Affiliations:** 1 Centre of Applied Health Research, University College London, 1-19 Torrington Place, London WC1E 6BT, UK; 2 Pensions and Annuity Group, Legal and General Assurance Society Limited, Surrey KT20 6EU, UK; 3 Institute of Psychology, Health and Society, University of Liverpool, Liverpool L69 3GB, UK

## Abstract

**Background:**

Our aims were to determine the pace of change in cardiovascular risk factors by age, gender and socioeconomic groups from 1994 to 2008, and quantify the magnitude, direction and change in absolute and relative inequalities.

**Methods:**

Time trend analysis was used to measure change in absolute and relative inequalities in risk factors by gender and age (16-54, ≥ 55 years), using repeated cross-sectional data from the Health Survey for England 1994-2008. Seven risk factors were examined: smoking, obesity, diabetes, high blood pressure, raised cholesterol, consumption of five or more daily portions of fruit and vegetables, and physical activity. Socioeconomic group was measured using the Index of Multiple Deprivation 2007.

**Results:**

Between 1994 and 2008, the prevalence of smoking, high blood pressure and raised cholesterol decreased in most deprivation quintiles. However, obesity and diabetes increased. Increasing absolute inequalities were found in obesity in older men and women (*p *= 0.044 and *p *= 0.027 respectively), diabetes in young men and older women (*p *= 0.036 and *p *= 0.019 respectively), and physical activity in older women (*p *= 0.025). Relative inequality increased in high blood pressure in young women (*p *= 0.005). The prevalence of raised cholesterol showed widening absolute and relative inverse gradients from 1998 onwards in older men (*p *= 0.004 and *p *≤ 0.001 respectively) and women (*p *≤ 0.001 and *p *≤ 0.001).

**Conclusions:**

Favourable trends in smoking, blood pressure and cholesterol are consistent with falling coronary heart disease death rates. However, adverse trends in obesity and diabetes are likely to counteract some of these gains. Furthermore, little progress over the last 15 years has been made towards reducing inequalities. Implementation of known effective population based approaches in combination with interventions targeted at individuals/subgroups with poorer cardiovascular risk profiles are therefore recommended to reduce social inequalities.

## Background

Coronary heart disease (CHD) mortality rates have substantially decreased since the 1970s in England, as in most Western populations. International studies suggest that 50-75% of the reductions in deaths from cardiac causes can be attributed to improvements in the major risk factors at population level (particularly smoking but also cholesterol and blood pressure levels), whereas the remaining 25-50% can be attributed to medical interventions [1-4].

However, outstanding issues remain when modelling past and future mortality trends in CHD. The most important concerns inequalities. Mortality from CHD is known to be inequitably distributed across socioeconomic groups [5,6]. Recent analysis in Scotland showed six-fold differentials in CHD mortality rates in young people living in the most and least deprived areas [7]. In England, narrowing of absolute inequalities in age adjusted CHD death rates from 1982 to 2006 coincided with slower relative rates of improvement in the most deprived areas [8].

Although downward trends in CHD mortality have been impressive, the slower relative rates of improvement in the most deprived quintiles show that the gains could have been larger than those observed had the gains been shared equally across all areas. Given the importance of risk factors in explaining population trends in CHD, it stands to reason that any change in the magnitude and/or direction of socioeconomic gradients in CHD mortality may be explained by parallel changes in risk factors [9]. However, evidence on changes in social inequalities in risk factors in England is limited. A prospective cohort study over a twenty year period assessed major risk factors only twice and did not include women or older men [10]. Yet monitoring the magnitude, direction and change in risk factors by social groups in the adult population as a whole may have powerful implications for present and future inequalities in CHD mortality.

Using data from the Health Survey for England (HSfE), we assessed the pace of change in seven cardiovascular risk factors by age, gender, and socioeconomic groups from 1994 to 2008 and monitored changes in absolute and relative inequalities. Both measures are essential: using relative measures alone fails to allow monitoring of changes in absolute risk factor levels across groups [11]. Furthermore, the size, direction and change in measures of inequality are associated with underlying levels of health. Relative inequalities tend to be larger when prevalence is low, whereas inequalities measured on an absolute scale are negligible at both very low and very high levels [12,13]. If levels of risk factor exposure decline across all groups (i.e. improve over time) declines in absolute inequalities (which are beneficial from the perspective of overall population health), may coincide with increasing inequalities on the relative scale. Guidance from the World Health Organization recommends that monitoring both absolute and relative inequalities is needed to provide a clear picture of health and its distribution across society, and, crucially, to assess policy impacts on health equity [14].

## Methods

### Population and study design

The Health Survey for England (HSfE), an annual nationwide health examination survey of the English non-institutional population, has been described in detail elsewhere [15]. Briefly, members of a stratified random household sample (drawn from the Postcode Address File) that is socio-demographically representative of the English population were invited to participate. The annual household response rate was approximately 78% in 1994, decreasing steadily to 64% in 2008. Data were collected at two visits. Firstly an interviewer's visit during which a questionnaire was administered and height and weight were measured. Secondly a visit from a trained nurse which included collection of blood samples, measurements of blood pressure and additional questioning including use of prescribed medication.

### Risk factor measurements

We obtained data on seven risk factors according to age, gender, deprivation quintiles, and survey year (Table 1). Current cigarette smoking status and obesity (BMI ≥ 30 kg/m^2^) were measured annually; high blood pressure was collected in all years except 1999 and 2004; reported fruit and vegetable consumption was recorded from 2001 onwards. A detailed module on cardiovascular disease and associated risk factors, including total cholesterol, diabetes, and physical activity was included in 1994, 1998, 2003, and 2006. Cholesterol and physical activity were also included in 2008. The physical activity questionnaire used in 1994 was not comparable to that used in later years and so our start year for estimating trends was 1998. Work-based activities were excluded from the summary measure of physical activity. Raised cholesterol was defined using a threshold of 5.0 mmol/l irrespective of whether respondents were currently taking lipid-lowering medication. This definition is consistent with usual Health Survey for England reporting which in turn reflects National Institute of Health and Clinical Excellence (NICE) guidelines [16]. High blood pressure was defined in terms of raised systolic blood pressure (≥ 140 mmHg) as it better predicts CHD [17]. We examined trends in raised cholesterol and blood pressure irrespective of medication use as our main objective was to assess whether absolute and/or relative inequalities had diminished or increased regardless of the underlying reason. (For the sample sizes in each year for the main interview, nurse visit, and blood samples see Additional files 1, 2 and 3).

**Table 1:** Risk factor definitions, availability and sample size

Risk factor	Description	Years	**Respondents (N)**^**a**^
***Self-reported measures***			
**Current cigarette smoking**	Self-reported status.	1994-2008	181 619
**Diabetes**	Those reporting diabetes that was doctor-diagnosed, excluding women who had only had diabetes during pregnancy.	1994,1998, 2003,2006	59 071
**Physical activity**	High levels defined as spending 30 minutes or more of moderate or vigorous activity on at least five days per week. No account was taken of exercise at work.	1998,2003, 2006,2008	58 184
**Fruit and vegetable consumption**	Portions per day. Healthy eating defined as consuming five or more portions per day.	2001-8	91 225
***Physical examination measurements***			
**Obesity**	Obesity defined as BMI 30 kg/m^2 ^or more.	1994-2008	161 663
**High blood pressure**	Calculated as the mean of the 2nd and 3rd readings for those who had not eaten, consumed alcohol or smoked in the 30 minutes prior to measurement. High blood pressure defined as SBP at or greater than 140 mmHg.	All years except 1999 and 2004^b^	117 631
**Raised cholesterol**	Raised cholesterol defined as total cholesterol at or above 5.0 mmol/l. Those who reported taking lipid lowering drugs were included.	1994,1998, 2003, 2006,2008	44 743

^a ^Adults aged 16 and over

**^b ^**Owing to small sample sizes survey data from 1997 onwards was pooled by merging two consecutive years

### Measure of socioeconomic circumstance

Socioeconomic circumstance was measured by the Index of Multiple Deprivation 2007 (IMD 2007). This is a composite index of relative deprivation at small area level (Lower Super Output Areas: LSOAs) based on seven domains of deprivation: income; employment; health deprivation and disability; education, skills and training; barriers to housing and services; crime and disorder, and living environment [18,19]. Deprivation indices developed prior to the introduction of IMD such as the Carstairs Index and Townsend Index were based solely on census data and compiled at the electoral ward level of geography. The advantage of using IMD is that it combines census data with other data sources which can be updated regularly over the inter-censal period. Furthermore, it is calculated at LSOA level whose boundaries, unlike electoral wards, remain fixed over time making IMD more suitable for measuring change over time. LSOAs have a mean population of 1,500 people and so are smaller on average than wards (average of 6,000). Using smaller areas increases the likelihood that populations are more homogenous - larger areas such as wards are more likely to group together populations which differ in levels of deprivation [20].

IMD was first introduced in 2004 (based on 2001 data) and has been updated in 2007 (based largely on 2005 data) and, most recently, in 2010 (using 2008 data). IMD scores are compiled using data from the 2001 Census and a variety of sources including from routine administrative returns to government departments (Health, Work and Pensions, HM Revenue & Customs, Children, Schools and Families, Communities and Local Government, Transport, Office for National Statistics) and non-governmental agencies (National Asylum Support Service, Prescribing Pricing Authority, Higher Education Statistics Agency and modelled estimates produced by Heriot-Watt University).

IMD 2007 scores of all LSOAs in England were grouped into quintiles, ranked in ascending order of deprivation score (Q1 most affluent; Q5 most deprived). The postcode address of responding households in each survey was linked to the LSOA and hence the corresponding deprivation quintile. 21 adults (< 0.02%) could not be linked and so were excluded from the analysis. Approximately one-fifth of the English population resides in each deprivation quintile. In comparison, 21.1% of survey respondents to the main interview lived in Q1 compared to 18.5% in Q5; equivalent figures for the nurse visit and blood samples were 22.2% and 17.0% and 22.5% and 16.6% [Additional files 1, 2 and 3].

### Statistical methods

Analyses were conducted separately for men and women stratified by age (16-54, ≥ 55 years). Data within these broad age bands were age standardised using ten year bands by the direct method using the European Standard Population as reference. Survey data from 2003 onwards were weighted for non-response, with different weights applied to the main interview, nurse visit, and blood samples. Non-response weights were not produced for data prior to 2003 due to good response rates in earlier surveys. The HSfE uses a clustered, stratified multistage sample design. To account for this complex design, 95% confidence intervals (CIs) were calculated using Stata version 11.1 (Stata Corp., College Station, Texas, USA).

#### Changes in risk factors over time

Two methods were used to estimate risk factor change. Firstly data from the first and last available year were used to estimate absolute change (i.e. percentage point differences for binary variables). Secondly log-binomial regression models were used to estimate annual change in prevalence ratios (PR) using the specific cardiovascular risk factor as the dependent variable with survey year and age as continuous independent variables. Two models were fitted to each quintile. The first model fitted the linear trend and so expressed annual change as a constant PR (i.e. estimated prevalence in year *t*/estimated prevalence in year *t-1*). The second model fitted linear and quadratic trends (i.e. allowing for acceleration or deceleration in the pace of change). Quadratic trends were examined for risk factors that had a (mostly) continuous data series. Quadratic terms not significant at the 1% level were removed from the model leaving just the linear trend.

#### Change in absolute inequalities

Linear regression models were used to estimate absolute differences in risk factor prevalence with the risk factor as the dependent variable and IMD, age, and survey year as the three independent variables. Three models were fitted. First, four indicator variables for IMD were used with the most affluent quintile (Q1) selected to act as the reference category (Model 1a). The four coefficients denoted the difference in prevalence between each quintile and Q1 (year and age adjusted). To present a more parsimonious model in the event of a linear relationship, we fitted an alternative model using IMD as a five category ordinal level variable ranging from 1 to 5 (Model 2a). The coefficient for IMD denoted the difference in prevalence for a one level (unit) increase in IMD quintile (year and age adjusted). Using a linear term means that a unit increase produces the same absolute difference in prevalence regardless of where that unit increase occurs along the five category ordinal scale. The *p*-value served as a test for linear trend (5% as the threshold for statistical significance).

We assessed change in absolute inequalities over time using the significance level of the coefficient(s) for an interaction term *IMD × survey year*, which was added to the model including IMD, age, and survey year as independent variables (Model 3a). IMD was represented by an ordinal level variable if Model 2a showed supportive evidence of a linear trend. In this case, the interaction was represented in the model by a single term. Four indicator variables represented IMD if Model 2a did not show a linear trend. In this case, an overall test of four terms was used to examine whether the absolute changes in prevalence between each quintile and Q1 were all jointly equal to zero (i.e. no trend interaction effects).

#### Change in relative inequalities

A similar procedure using log-binomial regression was used to estimate relative inequalities [21]. Model 1b (IMD represented by four indicator variables) estimated the PR between each quintile and Q1 (year and age adjusted). The same ordinal level variable as above was used to examine any linear relationship (Model 2b) with the single term denoting change in the PR for a unit increase in IMD (year and age adjusted) and its *p*-value acted as a test of linear trend. An *IMD × survey year *interaction term(s) was used to assess change in relative inequalities over time (Model 3b) with the results from Model 2b determining whether IMD was represented in the model by a single ordinal level variable (linear trend) or four indicator variables (non-linearity).

In summary, therefore, we computed 56 tests of change in inequalities over time: gender (2) × age-group (2) × risk factors (7) × inequality measure (2).

#### Sensitivity analyses - IMD minus the health domain

The IMD includes a health component which may lead to overestimation of the association between area-based deprivation and risk factors. We created an 'IMD-minus-health' quintile variable by standardising and exponentially transforming the six non-health domains and computing a non-health score by reallocating the health domain weight across the other domains in proportion to their original weights [18,19,22] and matching this variable to the survey data. Our analyses showed that excluding the health domain had little practical effect on the magnitude of absolute and relative inequalities with no systematic pattern in the differences, and thus the results of using the full IMD are presented here. (For results using the IMD measure excluding the health domain see Additional files 4 and 5).

## Results

### Trends in cardiovascular risk factors

**Figure 1:**
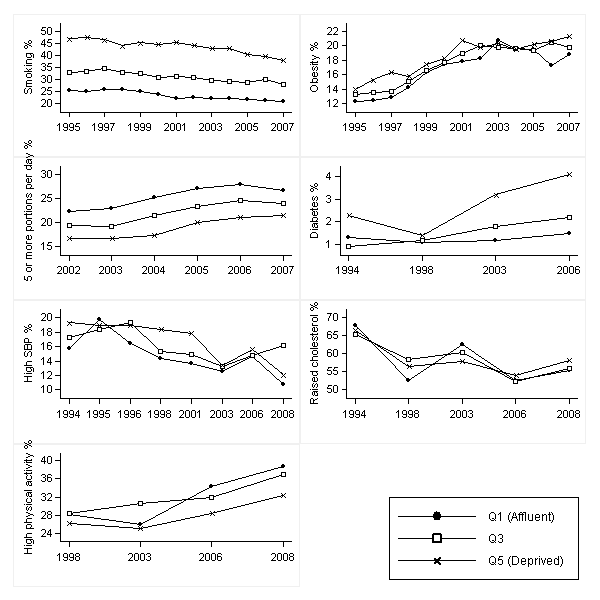
**Trends in age-standardised risk factors over 1994-2008 by IMD quintiles in men aged 16-54 years**. Smoothed estimates based on three-year moving averages for smoking, obesity and fruit and vegetable consumption. Smoothed estimates for high blood pressure obtained by merging two consecutive years (from 1997 onwards). High blood pressure defined as SBP ≥ 140 mmHg; raised cholesterol as total cholesterol ≥ 5.0 mmol/l; and high physical activity as meeting the recommendations of participating in moderate or vigorous activities for at least 30 min duration on at least five days per week (excluding work-based activities)

**Figure 2:**
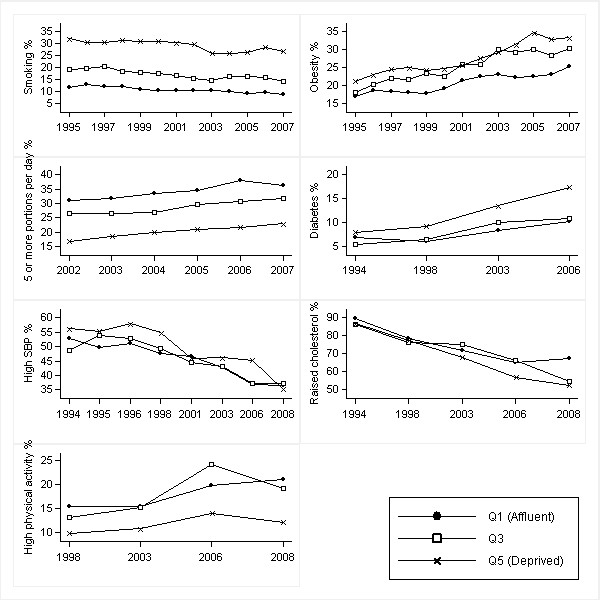
**Trends in age-standardised risk factors over 1994-2008 by IMD quintiles in men aged ≥ 55 years**. Smoothed estimates based on three-year moving averages for smoking, obesity and fruit and vegetable consumption. Smoothed estimates for high blood pressure obtained by merging two consecutive years (from 1997 onwards). High blood pressure defined as SBP ≥ 140 mmHg; raised cholesterol as total cholesterol ≥ 5.0 mmol/l; and high physical activity as meeting the recommendations of participating in moderate or vigorous activities for at least 30 min duration on at least five days per week (excluding work-based activities)

**Figure 3:**
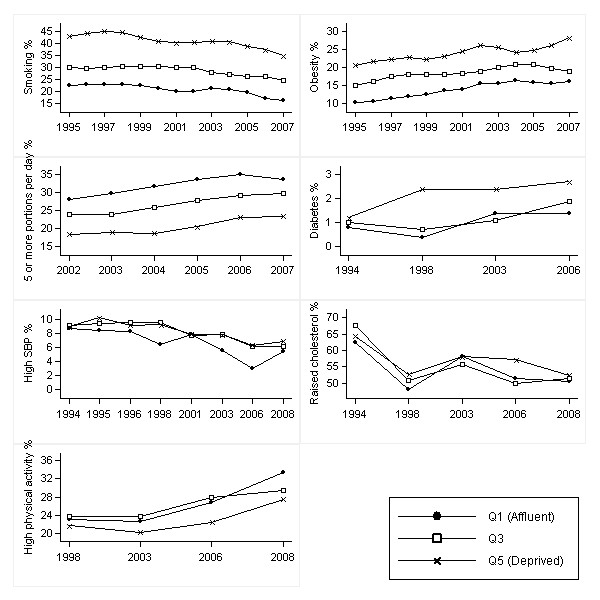
**Trends in age-standardised risk factors over 1994-2008 by IMD quintiles in women aged 16-54 years**. Smoothed estimates based on three-year moving averages for smoking, obesity and fruit and vegetable consumption. Smoothed estimates for high blood pressure obtained by merging two consecutive years (from 1997 onwards). High blood pressure defined as SBP ≥ 140 mmHg; raised cholesterol as total cholesterol ≥ 5.0 mmol/l; and high physical activity as meeting the recommendations of participating in moderate or vigorous activities for at least 30 min duration on at least five days per week (excluding work-based activities)

**Figure 4:**
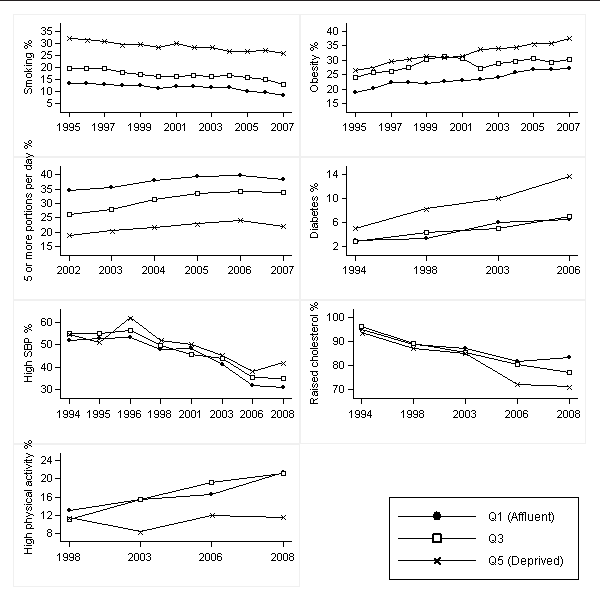
**Trends in age-standardised risk factors over 1994-2008 by IMD quintiles in women aged ≥ 55 years**. Smoothed estimates based on three-year moving averages for smoking, obesity and fruit and vegetable consumption. Smoothed estimates for high blood pressure obtained by merging two consecutive years (from 1997 onwards). High blood pressure defined as SBP ≥ 140 mmHg; raised cholesterol as total cholesterol ≥ 5.0 mmol/l; and high physical activity as meeting the recommendations of participating in moderate or vigorous activities for at least 30 min duration on at least five days per week (excluding work-based activities)

Figures 1, 2, 3 and 4 show age-standardised risk factor trends by gender, age group, and deprivation quintile. Estimates of absolute change are shown in Additional files 6, 7, 8 and 9; estimates of annual change in prevalence ratios in Additional files 10 and 11.

#### Overall change in risk factor levels, England 1994-2008

The prevalence of smoking, high blood pressure, and raised cholesterol decreased from 1994 to 2008. Smoking prevalence fell by 4.6% and 4.9% in young and old men respectively. The percentage of smokers fell by 6.1% in older women and was unchanged in young women until 2002 and then declined thereafter (7.5% reduction). The prevalence of high blood pressure fell by 3.5% in young men and by 14.4% in older men; equivalent figures in women were 3.2% and 19.6%. The prevalence of raised cholesterol fell by 9.7% and 29.1% in young and old men; equivalent figures in women were 11.6% and 18.0%. Levels of both self-reported physical activity and consumption of five or more daily portions of fruit and vegetables increased in men and women in both age groups. However, obesity and diabetes increased. Obesity prevalence increased by 8.4% in young men and by 7.6% in both young and old women; a 14.5% increase occurred in older men. The prevalence of diabetes increased by 1% and 5% in the youngest and oldest age groups [see Additional files 6, 7, 8 and 9].

#### Change in risk factor levels by IMD quintiles

The direction and pace of risk factor change within each IMD quintile largely mirrored those in England as a whole. There were, however, a number of exceptions. Annual falls in smoking prevalence were significant across all quintiles with the exception of older women in Q4. Declines in the prevalence of raised cholesterol were higher for older women in the most deprived quintiles. In the older age group, all quintiles showed increases in the prevalence of obesity with the exception of women in Q2. The low percentage achieving high levels of physical activity remained unchanged in older men in Q4 and Q5 and in older women in Q5. Since 2001 the percentage consuming five or more daily portions of fruit and vegetables remained unchanged in older men in Q2 and in older women in Q5 [see Additional files 6, 7, 8, 9, 10 and 11].

### Socioeconomic gradients in risk factor levels

Tests of linear association (Models 2a and 2b in Tables 2 and 3) showed that, after adjusting for age and survey year, risk factors had clear socioeconomic gradients in both absolute and relative terms: risk factor profiles being most favourable in Q1 (most affluent) and progressively worse along the IMD scale to Q5 (most deprived). The prevalence of raised cholesterol was an exception showing no linear relationship in the youngest age group and an inverse gradient in the oldest. Adjusting for survey year and age, a unit increase in IMD was associated with an absolute decline in the prevalence of raised cholesterol of 1.8% in older men and 1.3% in older women.

**Table 2:** Absolute and relative inequalities in cardiovascular risk factors in men (95% CIs in parentheses) by age-group

	Current smoking	Obesity	Diabetes	High levels of physical activity	High blood pressure (SBP ≥ 140 mmHg)	Raised cholesterol (TC ≥ 5 mmol/l)	≥ 5 portions of fruit & vegetables
**16-54**							
**Absolute difference**							
*Model 1a^§^*							
Q1 (reference)	0	0	0	0	0	0	0
Q2	2.8 (1.5,4.1)	0.4 (-0.6,1.5)	0.4 (-0.2,0.9)	1.1 (-1.4,3.7)	1.0 (-0.2,2.1)	0.3 (-2.3,2.9)	-2.5 (-4.3,-0.7)
Q3	7.4 (6.0,8.7)	0.9 (-0.1,2.0)	0.3 (-0.3,0.8)	0.1 (-2.4,2.6)	1.4 (0.2,2.7)	0.5 (-2.1,3.0)	-3.1 (-4.9,-1.3)
Q4	12.8 (11.4,14.2)	3.1 (2.0,4.2)	0.5 (0.0,1.1)	-0.5 (-3.0,2.0)	0.9 (-0.3,2.2)	-0.5 (-3.1,2.1)	-2.8 (-4.7,-1.0)
Q5	20.2 (18.7,21.6)	1.9 (0.8,3.0)	1.5 (0.8,2.1)	-3.9 (-6.3,-1.4)	2.2 (0.9,3.5)	0.8 (-1.9,3.5)	-5.9 (-7.8,-4.0)
*Model 2a^†^*	5.0 (4.7,5.3)	0.7 (0.4,0.9)	0.3 (0.1,0.5)	-0.9 (-1.5,-0.3)	0.4 (0.2,0.7)	0.1 (-0.5,0.7)	-1.2 (-1.6,-0.8)
*Model 3a^‡^*	*p *= 0.249	*p *= 0.555	*p *= 0.036	*p *= 0.340	*p *= 0.490	*p *= 0.709^|^	*p *= 0.709
**Relative (PR)**							
*Model 1b^§§^*							
Q1 (reference)	1	1	1	1	1	1	1
Q2	1.12 (1.06,1.18)	1.03 (0.97,1.10)	1.31 (0.90,1.92)	1.03 (0.95,1.12)	1.06 (0.98,1.15)	0.97 (0.94,1.00)	0.90 (0.84,0.97)
Q3	1.31 (1.25,1.38)	1.06 (1.00,1.13)	1.22 (0.83,1.80)	1.01 (0.93,1.09)	1.09 (1.01,1.18)	0.98 (0.95,1.01)	0.87 (0.81,0.94)
Q4	1.54 (1.47,1.61)	1.20 (1.13,1.28)	1.44 (0.98,2.10)	0.99 (0.91,1.07)	1.06 (0.98,1.15)	0.96 (0.93,0.99)	0.89 (0.82,0.96)
Q5	1.84 (1.76,1.93)	1.12 (1.05,1.20)	2.19 (1.54,3.13)	0.89 (0.81,0.96)	1.16 (1.07,1.26)	0.98 (0.95,1.01)	0.76 (0.70,0.83)
*Model 2b^††^*	1.17 (1.16,1.18)	1.04 (1.03,1.05)	1.19 (1.09,1.30)	0.97 (0.96,0.99)	1.03 (1.01,1.05)	1.00 (0.99,1.00)	0.95 (0.93,0.96)
*Model 3b^‡‡^*	*p *= 0.410	*p *= 0.124	*p *= 0.214	*p *= 0.680	*p *= 0.756	*p *= 0.737^|^	*p *= 0.781

**≥ 55 years**							
**Absolute difference**							
*Model 1a^§ ^*							
Q1 (reference)	0	0	0	0	0	0	0
Q2	3.0 (1.8,4.1)	3.3 (1.8,4.9)	-0.7 (-2.4,0.9)	0.2 (-2.2,2.6)	-0.9 (-3.0,1.2)	0.4 (-2.4,3.1)	0.0 (-2.3,2.3)
Q3	6.5 (5.3,7.8)	3.9 (2.3,5.5)	0.3 (-1.4,2.0)	0.1 (-2.3,2.5)	0.1 (-2.0,2.3)	-2.6 (-5.5,0.3)	-4.1 (-6.5,-1.8)
Q4	11.5 (10.1,12.9)	5.6 (3.9,7.3)	1.0 (-0.9,2.9)	-1.1 (-3.6,1.4)	1.8 (-0.5,4.0)	-6.5 (-9.6,-3.4)	-9.0 (-11.4,-6.7)
Q5	19.0 (17.5,20.5)	6.6 (4.8,8.3)	4.1 (1.9,6.3)	-6.2 (-8.6,-3.8)	4.0 (1.6,6.4)	-5.6 (-8.8,-2.3)	-13.1 (-15.5,-10.7)
*Model 2a^†^*	4.6 (4.3,4.9)	1.6 (1.2,2.0)	1.0 (0.5,1.4)	-1.3 (-1.9,-0.8)	1.0 (0.5,1.5)	-1.8 (-2.5,-1.1)	-3.5 (-4.0,-3.0)
*Model 3a^‡^*	*p *= 0.716	*p *= 0.044	*p *= 0.080	*p *= 0.229	*p *= 0.294	*p *= 0.004	*p *= 0.153
**Relative (PR)**							
*Model 1b^§§^*							
Q1 (reference)	1	1	1	1	1	1	1
Q2	1.29 (1.17,1.42)	1.16 (1.09,1.25)	0.91 (0.73,1.14)	1.01 (0.89,1.15)	0.98 (0.94,1.03)	1.00 (0.97,1.03)	1.00 (0.93,1.07)
Q3	1.62 (1.48,1.78)	1.19 (1.11,1.28)	1.04 (0.84,1.28)	1.00 (0.88,1.14)	1.00 (0.96,1.05)	0.98 (0.95,1.02)	0.88 (0.81,0.94)
Q4	2.10 (1.92,2.30)	1.27 (1.19,1.37)	1.13 (0.90,1.41)	0.94 (0.81,1.08)	1.04 (0.99,1.09)	0.94 (0.90,0.98)	0.73 (0.67,0.79)
Q5	2.81 (2.58,3.06)	1.32 (1.23,1.42)	1.52 (1.23,1.88)	0.65 (0.55,0.78)	1.09 (1.04,1.14)	0.95 (0.92,0.99)	0.60 (0.55,0.67)
*Model 2b^††^*	1.30 (1.27,1.32)	1.07 (1.05,1.08)	1.12 (1.06,1.18)	0.92 (0.89,0.96)	1.02 (1.01,1.03)	0.98 (0.98,0.99)	0.88 (0.87,0.90)
*Model 3b^‡‡^*	*p *= 0.016	*p *= 0.519	*p *= 0.394	*p *= 0.533	*p *= 0.562	*p *≤ 0.001	*p *= 0.032

PR: Prevalence ratio

Q1 = most affluent; Q5 = most deprived

^§ ^*Model 1a: *Percentage point (p.p) difference between IMD quintile and Q1 (adjusted for year and age). Linear regression model: year + age + Q2 + Q3 + Q4 + Q5

^† ^*Model 2a: *p.p difference for unit increase in IMD (fitted as ordinal level variable ranging from 1 to 5). Linear regression model: year + age + IMD. *p *from the model served as test of linear trend (statistical significance of absolute difference in p.p when moving from one ordinal category to one immediately higher). *p *≤ 0.05 if the 95% CIs do not include 0

^‡ ^*Model 3a: p *shown for interaction term testing change in absolute inequality over time. Linear regression model: year + age + IMD + (year × IMD). (^|^IMD fitted as 4 indicator variables; otherwise fitted as ordinal)

^§§ ^*Model 1b: *PR between IMD quintile and Q1 (adjusted for year and age). Log-binomial regression model: year + age + Q2 + Q3 + Q4 + Q5

^†† ^*Model 2b: *PR for unit increase in IMD (fitted as an ordinal level variable). Log-binomial regression model: year + age + IMD. *p *served as test for linear trend (change in PR when moving from one ordinal category to one immediately higher). *p *≤ 0.05 if the 95% CIs do not include 1

^‡‡ ^*Model 3b: p *shown for interaction term testing change in relative inequality over time. Log-binomial regression model: year + age + IMD + (year × IMD). (^|^IMD fitted as 4 indicator variables; otherwise fitted as ordinal)

**Table 3:** Absolute and relative inequalities in cardiovascular risk factors in women (95% CIs in parentheses) by age-group

	Current smoking	Obesity	Diabetes	High levels of physical activity	High blood pressure (SBP ≥ 140 mmHg)	Raised cholesterol (TC ≥ 5 mmol/l)	≥ 5 portions of fruit & vegetables
**16-54**							
**Absolute difference**							
*Model 1a^§^*							
Q1 (reference)	0	0	0	0	0	0	0
Q2	3.0 (1.9,4.2)	1.8 (0.8,2.7)	-0.2 (-0.6,0.3)	0.2 (-1.9,2.3)	0.2 (-0.5,0.9)	1.9 (-0.9,4.7)	-1.7 (-3.5,0.1)
Q3	8.1 (6.9,9.3)	4.4 (3.4,5.4)	0.2 (-0.3,0.6)	-0.3 (-2.4,1.8)	1.5 (0.8,2.3)	1.7 (-1.1,4.4)	-3.9 (-5.7,-2.1)
Q4	13.8 (12.6,15.0)	6.9 (5.9,8.0)	0.2 (-0.3,0.6)	-0.8 (-2.9,1.2)	1.7 (1.0,2.5)	0.6 (-2.1,3.4)	-5.9 (-7.6,-4.1)
Q5	20.1 (18.8,21.4)	10.5 (9.4,11.5)	1.1 (0.6,1.7)	-3.5 (-5.6,-1.5)	1.6 (0.8,2.5)	3.2 (0.4,6.0)	-10.4 (-12.2,-8.7)
*Model 2a^†^*	5.1 (4.8,5.4)	2.6 (2.4,2.8)	0.3 (0.1,0.4)	-0.8 (-1.2,-0.3)	0.5 (0.3,0.7)	0.5 (-0.1,1.1)	-2.5 (-2.9,-2.1)
*Model 3a^‡^*	*p *= 0.116	*p *= 0.611	*p *= 0.222	*p *= 0.082	*p *= 0.050	*p *= 0.249^|^	*p *= 0.991
**Relative (PR)**							
*Model 1b^§§^*							
Q1 (reference)	1	1	1	1	1	1	1
Q2	1.15 (1.09,1.21)	1.13 (1.06,1.21)	0.85 (0.54,1.33)	1.00 (0.93,1.09)	1.02 (0.92,1.14)	1.02 (0.98,1.05)	0.94 (0.89,1.00)
Q3	1.39 (1.32,1.46)	1.32 (1.24,1.41)	1.16 (0.77,1.77)	0.99 (0.91,1.07)	1.22 (1.10,1.34)	1.02 (0.98,1.06)	0.87 (0.82,0.93)
Q4	1.67 (1.59,1.75)	1.51 (1.42,1.61)	1.16 (0.75,1.79)	0.97 (0.89,1.05)	1.24 (1.12,1.37)	1.00 (0.96,1.04)	0.81 (0.76,0.86)
Q5	1.96 (1.87,2.06)	1.77 (1.67,1.88)	2.17 (1.49,3.17)	0.87 (0.80,0.94)	1.24 (1.12,1.38)	1.05 (1.01,1.09)	0.66 (0.61,0.71)
*Model 2b^††^*	1.19 (1.18,1.20)	1.16 (1.14,1.17)	1.23 (1.12,1.36)	0.97 (0.95,0.99)	1.06 (1.04,1.09)	1.00 (0.99,1.02)	0.91 (0.90,0.92)
*Model 3b)^‡‡^*	*p *= 0.187	*p *= 0.029	*p *= 0.998	*p *= 0.151	*p *= 0.005	*p *= 0.542^|^	*p *= 0.231

**≥ 55 years**							
**Absolute difference**							
*Model 1a^§^*							
Q1 (reference)	0	0	0	0	0	0	0
Q2	1.8 (0.7,3.0)	3.1 (1.6,4.6)	-0.4 (-1.5,0.8)	-0.9 (-3.0,1.2)	0.6 (-1.3,2.5)	-0.5 (-2.4,1.4)	-2.3 (-4.4,-0.1)
Q3	5.4 (4.2,6.6)	4.7 (3.1,6.2)	0.0 (-1.1,1.2)	0.0 (-2.1,2.2)	1.7 (-0.3,3.7)	-1.0 (-3.0,1.0)	-6.1 (-8.3,-4.0)
Q4	9.5 (8.2,10.8)	8.0 (6.4,9.7)	2.7 (1.3,4.1)	-2.4 (-4.6,-0.3)	2.0 (-0.1,4.0)	-4.4 (-6.8,-2.1)	-9.6 (-11.9,-7.4)
Q5	17.4 (15.9,18.9)	8.9 (7.1,10.6)	4.4 (2.8,6.0)	-5.7 (-7.9,-3.5)	4.2 (2.0,6.3)	-4.7 (-7.3,-2.2)	-16.0 (-18.2,-13.8)
*Model 2a^†^*	4.2 (3.9,4.5)	2.3 (1.9,2.7)	1.2 (0.8,1.5)	-1.3 (-1.7,-0.8)	1.0 (0.5,1.4)	-1.3 (-1.9,-0.8)	-3.9 (-4.4,-3.4)
*Mode13a^‡^*	*p *= 0.431	*p *= 0.027	*p *= 0.019	*p *= 0.025	*p *= 0.251	*p *≤ 0.001	*p *= 0.534
**Relative (PR)**							
*Model 1b^§§^*							
Q1 (reference)	1	1	1	1	1	1	1
Q2	1.15 (1.05,1.26)	1.13 (1.06,1.20)	0.92 (0.71,1.19)	0.95 (0.84,1.09)	1.01 (0.97,1.06)	1.00 (0.98,1.02)	0.94 (0.89,1.00)
Q3	1.46 (1.34,1.59)	1.20 (1.13,1.28)	1.01 (0.79,1.29)	1.01 (0.88,1.14)	1.04 (0.99,1.08)	1.00 (0.98,1.02)	0.83 (0.78,0.89)
Q4	1.81 (1.66,1.96)	1.35 (1.27,1.43)	1.57 (1.24,1.98)	0.86 (0.74,0.99)	1.04 (1.00,1.09)	0.97 (0.94,0.99)	0.74 (0.69,0.80)
Q5	2.48 (2.29,2.69)	1.39 (1.30,1.48)	1.95 (1.54,2.45)	0.66 (0.55,0.78)	1.09 (1.04,1.14)	0.97 (0.94,0.99)	0.56 (0.51,0.61)
*Model 2b^††^*	1.27 (1.24,1.29)	1.09 (1.07,1.10)	1.22 (1.15,1.29)	0.92 (0.89,0.95)	1.02 (1.01,1.03)	0.99 (0.99,1.00)	0.88 (0.86,0.89)
*Model 3b^‡‡^*	*p *= 0.088	*p *= 0.208	*p *= 0.639	*p *= 0.172	*p *= 0.064^∫^	*p *≤ 0.001	*p *= 0.065

PR: Prevalence ratio

Q1 = most affluent; Q5 = most deprived

^§ ^*Model 1a: *Percentage point (p.p) difference between IMD quintile and Q1 (adjusted for year and age). Linear regression model: year + age + Q2 + Q3 + Q4 + Q5

^† ^*Model 2a: *p.p difference for unit increase in IMD (fitted as ordinal level variable ranging from 1 to 5). Linear regression model: year + age + IMD. *p *from the model served as test of linear trend (statistical significance of absolute difference in p.p when moving from one ordinal category to one immediately higher). *p *≤ 0.05 if the 95% CIs do not include 0

^‡ ^*Model 3a: p *shown for interaction term testing change in absolute inequality over time. Linear regression model: year + age + IMD + (year × IMD). (^|^IMD fitted as 4 indicator variables; otherwise fitted as ordinal)

^§§ ^*Model 1b: *PR between IMD quintile and Q1 (adjusted for year and age). Log-binomial regression model: year + age + Q2 + Q3 + Q4 + Q5

^†† ^*Model 2b: *PR for unit increase in IMD (fitted as an ordinal level variable). Log-binomial regression model: year + age + IMD. *p *served as test for linear trend (change in PR when moving from one ordinal category to one immediately higher). *p *≤ 0.05 if the 95% CIs do not include 1

^‡‡ ^*Model 3b: p *shown for interaction term testing change in relative inequality over time. Log-binomial regression model: year + age + IMD + (year × IMD). (^|^IMD fitted as 4 indicator variables; otherwise fitted as ordinal)

∫ Model fitted using Poisson regression due to log-binomial regression failing to converge

### Changes in absolute and relative inequalities

A total of 56 tests of change in inequalities over time are shown in Tables 2 and 3 for men and women respectively. Results for change in absolute inequalities are shown by Models 3a; change in relative inequalities by Models 3b.

Four tests - the prevalence of raised cholesterol in young men and women - showed no change over time, i.e. no association with IMD. No change in inequalities occurred in 38 tests; statistically significant changes were found in 14. Five tests showed increasing absolute inequalities in obesity in older men and women, diabetes in young men and older women, and physical activity in older women. Three tests showed increasing relative inequalities in obesity in young women and in smoking and healthy eating in older men. Both absolute and relative inequality increased in high blood pressure in young women. Four tests for the prevalence of raised cholesterol showed widening absolute and relative inverse gradients from 1998 onwards in older men and women.

Obesity trends in older men and women showed increasing absolute inequalities (*p *= 0.044 and *p *= 0.027 respectively) reflecting larger absolute increases in prevalence in deprived areas. For example, the prevalence of obesity in older women increased in absolute terms by 10.2% in Q1 and 14.3% in Q5. Relative to baseline, however, obesity levels in 2008 were approximately 57% higher in both groups [Figures 2 and 4: Additional files 7 and 9].

Widening absolute inequalities occurred in diabetes in older women (*p *= 0.019). Although diabetes prevalence increased in all IMD quintiles, it increased by 8.6% in Q5 compared to just 3.4% in Q1, resulting in the absolute difference between Q5 and Q1 increasing from 2.0% to 7.3% from 1994 to 2006 [Figure 4: Additional file 9]. Widening absolute inequalities (present in 2003 and 2006) occurred in diabetes in young men (*p *= 0.036). For example, the absolute difference between Q5 and Q1 increased from 1.0% in 1994 to 2.6% in 2006 reflecting an increase of 1.9% in Q5 and a negligible increase of 0.2% in Q1 [Figure 1: Additional file 6].

Widening absolute inequalities (no gradient in 1998; present in 2003, 2006 and 2008) occurred in physical activity in older women (*p *= 0.025). For example, the absolute difference in the percentage achieving recommended levels between Q1 and Q5 increased from 1.6% in 1998 to 10.0% in 2008 reflecting an 8.5% improvement in absolute terms in Q1 but a negligible increase of 0.1% in Q5 [Figure 4: Additional file 9].

The prevalence of raised cholesterol showed widening (from 1998) absolute and relative *inverse *gradients in older men and women (men: *p *= 0.004 for absolute inequality and *p *≤ 0.001 relative inequality; women: *p *≤ 0.001 for absolute and relative inequality). In older men, the absolute difference in the prevalence of raised cholesterol between Q1 and Q5 increased from 3.0% in 1994 to 14.7% in 2008 (an increase in the PR of 1.03 to 1.28) [Figure 2: Additional file 7]. The absolute difference in older women similarly increased from 1.5% to 12.2% (PR increase from 1.02 to 1.17) [Figure 4: Additional file 9]. In older men and women, falls in the prevalence of raised cholesterol (in both absolute and relative terms) were higher in most deprived areas.

Widening relative inequalities in smoking in older men (*p *= 0.016) reflect larger falls in absolute levels in the most affluent quintiles particularly in 2008 (and so should be viewed with some caution). Relative inequality increased in high blood pressure in young women (*p *= 0.005) while absolute inequality marginally rose (*p *= 0.050). These results must be interpreted with reference to the low prevalence levels at baseline (< 10%) and their decline since 1994 across all groups. In this situation, absolute measures of inequality are inevitably small whilst relative measures are likely to be high. The marginal increase in absolute inequality suggests larger falls in elevated blood pressure in most affluent areas: the absolute difference between Q5 and Q1 increased from 0.2% in 1994 to 1.7% in 2008 [Figure 3: Additional file 8].

## Discussion

Between 1994 and 2008 significant reductions in the prevalence of smoking, high blood pressure and raised cholesterol occurred in all deprivation quintiles and levels of physical activity and consumption of fruit and vegetables increased. However, obesity and diabetes increased. Risk factors showed clear social gradients with profiles being most favourable in affluent areas. Absolute inequalities in smoking have not reduced while absolute inequalities in obesity have increased in older people. In older women, absolute inequalities increased in diabetes and physical activity.

### Comparisons with other studies

Three recent reports showed similar trends in Western high-income countries since 1980: decreases in systolic blood pressure (SBP) and total cholesterol (TC) coinciding with increases in BMI/obesity [23-25]. Recent trends in England show gradual declines in smoking [26], falls in blood pressure [27,28] and cholesterol [29,30], small increases in fruit and vegetable consumption [31] and sport/exercise participation [32] but gradual increases in obesity [33] and diabetes [34,35]. Strong socioeconomic gradients have been reported in smoking [26], physical activity [36], fruit and vegetable consumption [31], in obesity in women [33] but not in lipid levels [29,30], much as in our study.

UK findings on changes in inequalities are mixed partly reflecting differences in definition, time period and study population. Between 1983 and 1994, relative (smoking, healthy eating and sports participation) and absolute (BMI and SBP) inequalities remained unchanged in adults in England [37]. Widening absolute and relative inequalities in diabetes were seen in women in England from 1994 to 2006, but not in men [35]. A marginal increase in absolute difference in smoking prevalence over 2001-03 and 2007-09 reflected slower declines in routine/manual occupations [38]. In middle-aged men, absolute inequalities in SBP and TC narrowed but widened in BMI over a twenty year period [10].

### Influence of policies on trends in risk factors and impact on inequalities

Risk factor reduction policies implemented in England include: (1) targeting "high-risk" individuals in primary care settings (e.g. financially incentivised screening and treatment of hypertension/dyslipidaemia with lifestyle advice and medications); (2) health promotional activities (e.g. a widely marketed mass media "5-a-day" fruit and vegetables programme); (3) "voluntary" targets for industry set by governments (e.g. salt reduction); and (4) whole-population based strategies (e.g. statutory regulation and environmental controls including smoke-free public places and cigarette taxation) which do *not *depend on an individual's resources.

Recent declines in blood pressure levels reflect changes in health behaviours and diet (e.g. through lower salt intake, lower tobacco consumption and higher physical activity), together with wider use of antihypertensive medication [39]. Salt intake levels have reduced by almost 1 g/day over 2001-08 in people aged 19-64 years, reflecting voluntary agreements with the food industry to reduce the salt content of processed foods, plus health promotional initiatives [40-42]. Hypertension management has also improved [27,28]. Increases in levels of BMI/obesity reflect trends towards larger portions and energy-dense foods, compounded by more sedentary lifestyles [43]. Rises in diabetes reflect increases in incidence plus improved case ascertainment [34,44,45].

The impact of risk factor reduction policies on UK health inequalities appears complex. Declines in smoking prevalence with persistent absolute inequalities probably reflect the combined effects of tobacco control policies including smoke free legislation introduced in July 2007 plus National Health Service smoking cessation services free at the point of use [46]. However, lower compliance and quit rates are reported in deprived groups [47]. Increasing absolute inequalities in obesity, diabetes and physical inactivity in older women probably reflect their strong associations [45].

### Strengths and limitations of the study

Our study included up-to-date information to 2008, large, nationally representative samples, high response rates, annual data, and standardised protocols to measure blood pressure, BMI and cholesterol. Presenting absolute and annual change informs interpretation of changes in inequalities. Absolute and relative measures of inequality were used to provide more complete detail. Study limitations include using self-reported measures which are prone to recall and response bias. Response rates were sub-optimal as elsewhere. However, data were weighted for non-response. Small sample sizes meant that our study lacked sufficient power to detect small changes in inequalities by subgroup e.g. in individuals with CHD. Other cardiovascular risk factors were not included (e.g. alcohol).

We chose IMD, a well-established marker of assigning socioeconomic circumstances based on area of residence for three main reasons. Firstly studies continue to show contextual associations between neighbourhood and health even after controlling for individual-level markers [48]. Residential deprivation is powerfully linked to health due to the influence of both composition (characteristics of individuals who live there) and context (features of the location itself) [49]. Area-based measures therefore may contribute additional socioeconomic information over and above that obtained from individual-level measures. Secondly area-based measures are particularly useful proxy measures of individual social position in older age groups [50,51]. Occupational-based schemas such as the UK National Statistics Socio-Economic Classification (NS-SEC) are not recommended for studying inequalities at older ages because of the large proportion that cannot be accurately classified. Third, stratifying Health Survey for England respondents by IMD enables us to examine whether recent changes in the magnitude and/or direction of socioeconomic gradients in CHD mortality may be explained by similar changes in its key risk factors. Area of residence (postcode) is recorded on death certificates and so area-based measures of deprivation can, to some extent, circumvent the difficulties in attributing socioeconomic status to older people and women [52,53]. Using identical stratifying variables to monitor changes in absolute and relative inequalities in both cardiovascular risk factors and death rates can shed important light on the possible potential drivers of longevity and inform discussions on possible future trends.

However, area-based measures are potentially subject to aggregation bias or "ecological fallacy" i.e. of assuming all individuals in an area possess similar characteristics [5]. The Index of Multiple Deprivation includes health-related data risking overestimation of the relationship between IMD and cardiovascular risk factors. However, a UK study has shown that removing the health domain from the overall index had little effect on categorisation of areas or the strength of relationship between area-based deprivation and health [22]. Negligible differences between the full IMD and 'IMD-minus-health' in the results of our study confirmed this finding.

One limitation of our study was that Health Survey for England respondents were assigned to the 2007 IMD measure (based on 2005 data) rather than assigned to a deprivation quintile compiled on data around the time of interview. This leads to the question of whether IMD 2007, which expresses the *relative *position of LSOAs to the average for England as a whole in 2005, is an accurate marker of deprivation across all survey years. A study using a comparable area-based indicator over 1991-2001 showed declines in absolute levels of deprivation accompanied by continuity in the relative deprivation status of wards [54]. Since 2001, the 2004, 2007 and 2010 IMD measures have retained broadly the same methodology, domains and indicators [19]. Our analyses showed reassuring stability in the relative position of LSOAs. Agreement between the 2004 and 2010 quintiles was 76% (kappa statistic = 0.70), indicating a good level of agreement.

## Conclusions

Between 1994 and 2008, smoking, blood pressure, and total cholesterol levels decreased in most deprivation quintiles. UK cohort studies have shown that these reductions played an important role in impressive declines in CHD related incidence and mortality despite concomitant increases in obesity and diabetes [55]. However, our analysis of Health Survey for England data over a 15 year period indicates little progress towards reducing inequalities. Despite a raft of policy initiatives, absolute inequalities in the prevalence of smoking have persisted while absolute inequalities in obesity have increased in older people.

Recent studies suggest that more socioeconomically disadvantaged groups will gain larger benefits, on an absolute scale, if unequally distributed risk factors are reduced proportionally across groups using whole-population based strategies [56,57]. However, although it is possible that policies such as cigarette taxation may particularly benefit more socioeconomically disadvantaged groups, the precise impact of other policies on the differential reduction of other major risk factors has not yet been established [56]. Furthermore, improvements in absolute but little progress in reducing relative inequalities would still leave groups at lower ends of the social hierarchy at a comparative disadvantage. Therefore, those evidence based population level strategies recommended but not implemented in England (e.g. food labelling, banning industrial transfats and mandatory changes to the food supply to halve the salt content of bread) should be introduced *in combination *with known effective interventions targeted at those at high-risk of cardiovascular events to achieve a narrowing of social inequalities.

## Abbreviations

BMI: Body mass index; CHD: Coronary heart disease; CI: Confidence interval; HSfE: Health Survey for England; IMD: Index of Multiple Deprivation 2007; LSOA: Lower Super Output Area; NHS: National Health Service; NS-SEC: National Statistics Socio-Economic Classification; PR: Prevalence ratio; SBP: Systolic blood pressure; TC: Total cholesterol; UCL: University College London; UK: United Kingdom.

## Competing interests

The authors declare that they have no competing interests.

## Authors' contributions

RR and SC: principal investigators of the project and SC is guarantor of the article; MB: overall coordinator of the project; SS: responsible for the design, analyses and drafting of the manuscript. All coauthors commented on the analysis and interpretation of the findings and approved the final version of the manuscript.

## Pre-publication history

The pre-publication history for this paper can be accessed here:

http://www.biomedcentral.com/1471-2458/12/129/prepub

## Supplementary Material

Additional file 1 **Health Survey for England 1994-2008 sample size (main interview), by gender, age and deprivation quintiles**. The table shows the sample sizes in each year for the main interview. Click here for file

Additional file 2 **Health Survey for England 1994-2008 sample size (nurse visit), by gender, age and deprivation quintiles**. The table shows the sample sizes in each year for the nurse visit. Click here for file

Additional file 3 **Health Survey for England 1994-2008 sample size (blood sample), by gender, age and deprivation quintiles**. The table shows the sample sizes in each year for the collection of blood samples. Click here for file

Additional file 4 **Absolute and relative inequalities in cardiovascular risk factors in men (95% CIs in parentheses) by age-group (using IMD excluding the health domain)**. The table shows absolute and relative inequalities in cardiovascular risk factors in men calculated using the 'IMD-minus-health domain' quintiles. Click here for file

Additional file 5 **Absolute and relative inequalities in cardiovascular risk factors in women (95% CIs in parentheses) by age-group (using IMD excluding the health domain)**. The table shows absolute and relative inequalities in cardiovascular risk factors in women calculated using the 'IMD-minus-health domain' quintiles. Click here for file

Additional file 6 **Absolute change in cardiovascular risk factors in men aged 16-54 years, by deprivation quintiles**. The table shows the absolute change in cardiovascular risk factors between the most affluent and most deprived fifths over 1994-2008 with accompanying 95% confidence intervals. Click here for file

Additional file 7 **Absolute change in cardiovascular risk factors in men aged ≥ 55 years, by deprivation quintiles**. The table shows the absolute change in cardiovascular risk factors between the most affluent and most deprived fifths over 1994-2008 with accompanying 95% confidence intervals. Click here for file

Additional file 8 **Absolute change in cardiovascular risk factors in women aged 16-54 years, by deprivation quintiles**. The table shows the absolute change in cardiovascular risk factors between the most affluent and most deprived fifths over 1994-2008 with accompanying 95% confidence intervals. Click here for file

Additional file 9 **Absolute change in cardiovascular risk factors in women aged ≥ 55 years, by deprivation quintiles**. The table shows the absolute change in cardiovascular risk factors between the most affluent and most deprived fifths over 1994-2008 with accompanying 95% confidence intervals. Click here for file

Additional file 10 **Annual change in cardiovascular risk factors in men, by deprivation quintile and age**. The table shows the annual change in cardiovascular risk factors for all deprivation fifths with accompanying 95% confidence intervals. Click here for file

Additional file 11 **Annual change in cardiovascular risk factors in women, by deprivation quintile and age**. The table shows the annual change in cardiovascular risk factors for all deprivation fifths with accompanying 95% confidence intervals. Click here for file
