# Supplementary material for: Persistent socioeconomic inequalities in cardiovascular risk factors in England over 1994-2008: A time-trend analysis of repeated cross-sectional data
Source: BMC Public Health. 2012 Feb 14;12:129. doi: 10.1186/1471-2458-12-129 (PMC3342910; doi:10.1186/1471-2458-12-129)
Supplement: Additional file 1 — Health Survey for England 1994-2008 sample size (main interview), by gender, age and deprivation quintiles. The table shows the sample sizes in each year for the main interview. [file 1471-2458-12-129-S1.PDF]

ADDITIONAL FILE 1. Health Survey for England 1994-2008 sample size (main interview), by gender, age and deprivation quintiles

	1994	1995	1996	1997	1998	1999	2000	2001	2002	2003	2004	2005	2006	2007	2008	Total
**Men 16-54**																
Q1	1001 (20.7%)	1073 (22.0%)	1036 (20.6%)	499 (18.9%)	995 (20.8%)	526 (22.6%)	466 (19.7%)	919 (20.7%)	708 (20.8%)	844 (19.9%)	381 (21.6%)	470 (21.5%)	730 (18.5%)	405 (20.7%)	824 (20.0%)	**10 877 (20.5%)**
Q2	974 (20.1%)	972 (19.9%)	1114 (22.1%)	554 (21.0%)	978 (20.5%)	467 (20.1%)	483 (20.4%)	943 (21.3%)	643 (20.4%)	812 (19.5%)	364 (21.2%)	413 (19.4%)	705 (18.8%)	327 (17.5%)	753 (18.5%)	**10 502 (20.1%)**
Q3	1084 (22.4%)	991 (20.3%)	1008 (20.0%)	617 (23.4%)	966 (20.2%)	470 (20.2%)	529 (22.4%)	801 (18.1%)	660 (19.1%)	768 (18.9%)	334 (19.4%)	406 (19.5%)	782 (21.4%)	402 (22.3%)	797 (19.9%)	**10 615 (20.4%)**
Q4	937 (19.3%)	908 (18.6%)	845 (16.8%)	485 (18.4%)	955 (20.0%)	422 (18.2%)	419 (17.7%)	934 (21.1%)	740 (21.5%)	893 (22.1%)	335 (20.5%)	464 (22.3%)	742 (20.6%)	354 (20.2%)	826 (21.4%)	**10 259 (20.0%)**
Q5	847 (17.5%)	938 (19.2%)	1028 (20.4%)	480 (18.2%)	883 (18.5%)	438 (18.9%)	468 (19.8%)	840 (18.9%)	599 (18.2%)	783 (19.6%)	280 (17.4%)	362 (17.3%)	746 (20.6%)	337 (19.1%)	802 (20.3%)	**9831 (19.1%)**
**Men ≥ 55**																
Q1	495 (21.4%)	508 (20.8%)	489 (20.0%)	216 (17.5%)	500 (21.0%)	274 (22.5%)	253 (19.9%)	579 (23.3%)	280 (24.5%)	658 (26.5%)	272 (22.8%)	603 (25.1%)	567 (21.9%)	303 (25.1%)	661 (24.3%)	**6658 (22.4%)**
Q2	488 (21.1%)	508 (20.8%)	521 (21.3%)	299 (24.2%)	535 (22.5%)	241 (19.8%)	336 (26.4%)	539 (21.7%)	261 (22.9%)	538 (21.8%)	307 (26.3%)	572 (22.3%)	578 (22.2%)	282 (22.9%)	607 (22.4%)	**6612 (22.3%)**
Q3	507 (21.9%)	483 (19.8%)	540 (22.1%)	259 (21.0%)	477 (20.0%)	253 (20.8%)	265 (20.8%)	480 (19.3%)	222 (19.5%)	484 (19.6%)	189 (16.6%)	497 (20.4%)	640 (24.9%)	250 (20.4%)	552 (20.5%)	**6098 (20.7%)**
Q4	408 (17.6%)	484 (19.8%)	379 (15.5%)	227 (18.4%)	461 (19.4%)	245 (20.1%)	207 (16.2%)	464 (18.7%)	204 (17.9%)	440 (18.1%)	212 (18.4%)	472 (19.3%)	441 (17.6%)	206 (17.1%)	465 (17.4%)	**5315 (18.1%)**
Q5	418 (18.0%)	456 (18.7%)	515 (21.1%)	232 (18.8%)	409 (17.2%)	206 (16.9%)	213 (16.7%)	420 (16.9%)	174 (15.2%)	343 (14.1%)	181 (15.9%)	331 (12.9%)	349 (13.5%)	173 (14.6%)	419 (15.5%)	**4839 (16.5%)**
**Women 16-54**																
Q1	1112 (19.8%)	1246 (21.9%)	1187 (20.2%)	587 (19.0%)	1215 (21.4%)	582 (20.7%)	553 (19.7%)	1118 (20.1%)	837 (20.3%)	1033 (20.0%)	479 (20.6%)	533 (20.0%)	883 (18.4%)	459 (19.4%)	1035 (20.2%)	**12 859 (20.2%)**
Q2	1109 (19.8%)	1076 (18.9%)	1220 (20.8%)	636 (20.6%)	1139 (20.1%)	565 (20.1%)	549 (19.6%)	1152 (20.7%)	784 (19.4%)	947 (18.8%)	453 (19.9%)	488 (18.6%)	932 (19.6%)	447 (19.9%)	908 (18.2%)	**12 405 (19.7%)**
Q3	1194 (21.3%)	1117 (19.6%)	1154 (19.7%)	661 (21.4%)	1147 (20.2%)	580 (20.6%)	596 (21.3%)	1055 (18.9%)	762 (18.6%)	925 (18.4%)	420 (18.8%)	460 (17.9%)	999 (21.6%)	469 (20.8%)	963 (19.4%)	**12 502 (19.9%)**
Q4	1106 (19.7%)	1113 (19.6%)	1050 (17.9%)	568 (18.4%)	1101 (19.4%)	517 (18.4%)	520 (18.5%)	1195 (21.4%)	989 (21.9%)	1145 (23.1%)	488 (22.1%)	598 (23.4%)	922 (20.3%)	455 (20.4%)	1023 (21.1%)	**12 790 (20.3%)**
Q5	1088 (19.4%)	1139 (20.0%)	1257 (21.4%)	640 (20.7%)	1069 (18.9%)	571 (20.3%)	586 (20.9%)	1056 (18.9%)	912 (19.8%)	997 (19.6%)	429 (18.7%)	525 (20.0%)	943 (20.2%)	448 (19.6%)	1057 (21.1%)	**12 717 (20.0%)**
**Women ≥ 55**																
Q1	570 (19.0%)	578 (19.2%)	589 (19.2%)	273 (17.4%)	625 (20.7%)	290 (20.6%)	302 (20.0%)	648 (21.1%)	328 (22.6%)	815 (25.9%)	337 (21.9%)	681 (22.9%)	686 (21.9%)	359 (23.8%)	784 (23.7%)	**7865 (21.3%)**
Q2	624	649	653	373	596	283	369	677	323	664	382	681	708	332	747	**8061**

	(20.8%)	(21.5%)	(21.3%)	(23.8%)	(19.7%)	(20.1%)	(24.4%)	(22.1%)	(22.2%)	(21.1%)	(25.0%)	(22.9%)	(22.8%)	(22.1%)	(22.5%)	**(21.9%)**
Q3	673	632	653	316	636	317	316	622	292	654	275	579	746	335	683	**7729**
	(22.4%)	(21.0%)	(21.3%)	(20.2%)	(21.1%)	(22.5%)	(20.9%)	(20.3%)	(20.1%)	(20.7%)	(18.0%)	(19.4%)	(24.0%)	(22.2%)	(20.7%)	**(21.1%)**
Q4	553	599	535	303	600	280	250	606	268	568	302	637	541	246	588	**6876**
	(18.4%)	(19.9%)	(17.4%)	(19.3%)	(19.9%)	(19.9%)	(16.5%)	(19.8%)	(18.5%)	(18.0%)	(19.4%)	(20.3%)	(17.8%)	(16.4%)	(17.8%)	**(18.7%)**
Q5	579	557	637	303	562	238	274	512	242	453	240	460	416	232	504	**6209**
	(19.3%)	(18.5%)	(20.8%)	(19.3%)	(18.6%)	(16.9%)	(18.1%)	(16.7%)	(16.7%)	(14.4%)	(15.6%)	(14.5%)	(13.6%)	(15.5%)	(15.3%)	**(17.1%)**

Notes: Respondent totals are presented unweighted; percentages show distribution across the deprivation fifths (unweighted 1994 to 2001, weighted for unequal selection probabilities in 2002 and 2005, weighted for non-response from 2003 onwards). (Q1 = most affluent, Q5 = most deprived).
