# Supplementary material for: Persistent socioeconomic inequalities in cardiovascular risk factors in England over 1994-2008: A time-trend analysis of repeated cross-sectional data
Source: BMC Public Health. 2012 Feb 14;12:129. doi: 10.1186/1471-2458-12-129 (PMC3342910; doi:10.1186/1471-2458-12-129)
Supplement: Additional file 2 — Health Survey for England 1994-2008 sample size (nurse visit), by gender, age and deprivation quintiles. The table shows the sample sizes in each year for the nurse visit. [file 1471-2458-12-129-S2.PDF]

**ADDITIONAL FILE 2** **Health Survey for England 1994-2008 sample size (nurse visit), by gender, age and deprivation quintiles**

| | 1994 | 1995 | 1996 | 1997 | 1998 | 2000 | 2001 | 2002 | 2003 | 2005 | 2006 | 2007 | 2008 | Total |
|---|---|---|---|---|---|---|---|---|---|---|---|---|---|---|
| **Men 16-54** | | | | | | | | | | | | | | |
| Q1 | 878 (20.8%) | 877 (22.9%) | 867 (21.6%) | 429 (20.1%) | 770 (21.8%) | 343 (21.1%) | 684 (22.6%) | 490 (23.9%) | 558 (21.4%) | 282 (24.1%) | 498 (20.3%) | 260 (21.9%) | 485 (20.3%) | **7421 (21.7%)** |
| Q2 | 858 (20.3%) | 796 (20.8%) | 883 (22.0%) | 463 (21.7%) | 750 (21.2%) | 341 (21.0%) | 674 (22.3%) | 418 (20.4%) | 519 (20.9%) | 215 (18.7%) | 409 (18.1%) | 216 (19.0%) | 463 (19.9%) | **7005 (20.6%)** |
| Q3 | 954 (22.6%) | 791 (20.7%) | 784 (19.6%) | 499 (23.4%) | 734 (20.7%) | 375 (23.1%) | 546 (18.1%) | 414 (20.2%) | 465 (19.3%) | 231 (22.3%) | 495 (22.9%) | 237 (22.4%) | 446 (19.9%) | **6971 (21.0%)** |
| Q4 | 821 (19.5%) | 694 (18.1%) | 681 (17.0%) | 388 (18.2%) | 685 (19.4%) | 271 (16.7%) | 616 (20.4%) | 440 (21.5%) | 519 (21.8%) | 233 (22.6%) | 410 (20.1%) | 194 (18.3%) | 453 (20.6%) | **6405 (19.5%)** |
| Q5 | 706 (16.7%) | 668 (17.5%) | 791 (19.7%) | 358 (16.8%) | 600 (17.0%) | 293 (18.1%) | 501 (16.6%) | 286 (14.0%) | 371 (16.6%) | 130 (12.3%) | 374 (18.6%) | 183 (18.4%) | 414 (19.4%) | **5675 (17.2%)** |
| **Men ≥ 55** | | | | | | | | | | | | | | |
| Q1 | 415 (21.1%) | 421 (21.3%) | 422 (21.1%) | 192 (18.7%) | 403 (21.7%) | 184 (19.8%) | 467 (25.7%) | 205 (25.4%) | 463 (26.8%) | 424 (27.3%) | 419 (23.6%) | 210 (26.2%) | 470 (26.0%) | **4695 (23.2%)** |
| Q2 | 423 (21.5%) | 426 (21.6%) | 438 (21.9%) | 255 (24.9%) | 422 (22.7%) | 245 (26.4%) | 411 (22.6%) | 193 (23.9%) | 370 (22.0%) | 380 (23.3%) | 390 (21.9%) | 180 (22.0%) | 402 (22.8%) | **4535 (22.6%)** |
| Q3 | 432 (22.0%) | 395 (20.0%) | 433 (21.6%) | 210 (20.5%) | 374 (20.1%) | 190 (20.5%) | 350 (19.2%) | 154 (19.1%) | 347 (20.8%) | 329 (20.7%) | 417 (24.1%) | 158 (19.8%) | 365 (20.6%) | **4154 (20.8%)** |
| Q4 | 332 (16.9%) | 384 (19.5%) | 302 (15.1%) | 191 (18.6%) | 366 (19.7%) | 157 (16.9%) | 330 (18.1%) | 136 (16.9%) | 288 (17.2%) | 287 (18.4%) | 288 (17.3%) | 140 (17.8%) | 297 (16.8%) | **3498 (17.6%)** |
| Q5 | 365 (18.6%) | 346 (17.5%) | 408 (20.4%) | 178 (17.3%) | 296 (15.9%) | 153 (16.5%) | 261 (14.3%) | 118 (14.6%) | 208 (13.3%) | 175 (10.3%) | 224 (13.0%) | 105 (14.1%) | 246 (13.9%) | **3083 (15.8%)** |
| **Women 16-54** | | | | | | | | | | | | | | |
| Q1 | 952 (20.2%) | 1008 (23.0%) | 991 (21.1%) | 496 (20.4%) | 930 (22.2%) | 416 (21.1%) | 802 (21.8%) | 569 (20.9%) | 681 (21.0%) | 333 (21.9%) | 598 (20.0%) | 280 (19.9%) | 647 (21.7%) | **8703 (21.3%)** |
| Q2 | 939 (19.9%) | 848 (19.3%) | 985 (21.0%) | 504 (20.7%) | 864 (20.6%) | 410 (20.8%) | 784 (21.3%) | 552 (20.3%) | 611 (20.3%) | 286 (19.2%) | 581 (20.4%) | 281 (21.2%) | 533 (18.5%) | **8178 (20.3%)** |
| Q3 | 1000 (21.2%) | 880 (20.0%) | 926 (19.7%) | 534 (22.0%) | 857 (20.5%) | 420 (21.4%) | 709 (19.3%) | 490 (18.0%) | 560 (18.7%) | 270 (19.4%) | 625 (22.6%) | 269 (20.5%) | 535 (18.7%) | **8075 (20.1%)** |
| Q4 | 945 (20.0%) | 823 (18.7%) | 838 (17.8%) | 437 (18.0%) | 799 (19.1%) | 345 (17.5%) | 754 (20.5%) | 610 (22.4%) | 662 (22.6%) | 307 (22.6%) | 510 (19.4%) | 254 (19.8%) | 573 (20.7%) | **7857 (19.8%)** |
| Q5 | 882 (18.7%) | 833 (19.0%) | 955 (20.3%) | 458 (18.9%) | 736 (17.6%) | 376 (19.1%) | 629 (17.1%) | 500 (18.4%) | 511 (17.4%) | 227 (16.8%) | 474 (17.6%) | 231 (18.5%) | 554 (20.4%) | **7366 (18.5%)** |
| **Women ≥ 55** | | | | | | | | | | | | | | |

| | | | | | | | | | | | | | | |
|---|---|---|---|---|---|---|---|---|---|---|---|---|---|---|
| Q1 | 456 (18.8%) | 478 (20.4%) | 480 (19.8%) | 235 (18.6%) | 489 (21.3%) | 218 (20.2%) | 489 (22.9%) | 241 (24.0%) | 556 (26.5%) | 454 (23.7%) | 476 (22.2%) | 241 (25.1%) | 530 (25.0%) | **5343 (21.9%)** |
| Q2 | 510 (21.1%) | 506 (21.6%) | 523 (21.6%) | 309 (24.5%) | 472 (20.6%) | 271 (25.1%) | 509 (23.9%) | 246 (24.5%) | 446 (21.2%) | 427 (23.2%) | 460 (22.2%) | 229 (24.0%) | 462 (22.1%) | **5370 (22.3%)** |
| Q3 | 553 (22.8%) | 471 (20.1%) | 514 (21.2%) | 252 (20.0%) | 473 (20.6%) | 229 (21.2%) | 441 (20.7%) | 193 (19.2%) | 435 (21.2%) | 386 (20.6%) | 522 (25.6%) | 209 (21.7%) | 443 (21.3%) | **5121 (21.3%)** |
| Q4 | 438 (18.1%) | 475 (20.2%) | 425 (17.6%) | 244 (19.3%) | 447 (19.5%) | 174 (16.1%) | 387 (18.1%) | 182 (18.1%) | 347 (17.5%) | 383 (19.7%) | 341 (17.2%) | 143 (14.9%) | 378 (18.1%) | **4364 (18.2%)** |
| Q5 | 465 (19.2%) | 418 (17.8%) | 479 (19.8%) | 223 (17.7%) | 414 (18.0%) | 187 (17.3%) | 308 (14.4%) | 144 (14.3%) | 266 (13.6%) | 244 (12.8%) | 251 (12.7%) | 132 (14.3%) | 281 (13.6%) | **3812 (16.2%)** |

*Notes:* Respondents with a valid blood pressure reading (including those taking drugs affecting blood pressure). Respondent totals are presented unweighted; percentages show distribution across the deprivation fifths (unweighted 1994 to 2001, weighted for unequal selection probabilities in 2002 and 2005, weighted for non-response from 2003 onwards). Totals may not sum due to rounding. Nurse visits not conducted in 1999 and 2004. (Q1 = most affluent, Q5 = most deprived).
