# Supplementary material for: Persistent socioeconomic inequalities in cardiovascular risk factors in England over 1994-2008: A time-trend analysis of repeated cross-sectional data
Source: BMC Public Health. 2012 Feb 14;12:129. doi: 10.1186/1471-2458-12-129 (PMC3342910; doi:10.1186/1471-2458-12-129)
Supplement: Additional file 3 — Health Survey for England 1994-2008 sample size (blood sample), by gender, age and deprivation quintiles. The table shows the sample sizes in each year for the collection of blood samples. [file 1471-2458-12-129-S3.PDF]

ADDITIONAL FILE 3. Health Survey for England 1994-2008 sample size (blood sample), by gender, age and deprivation quintiles

	1994	1998	2003	2006	2008	Total
**Men 16-54**						
Q1	772 (21.2%)	709 (21.7%)	503 (19.6%)	429 (19.1%)	418 (20.5%)	**2831 (20.5%)**
Q2	751 (20.6%)	695 (21.3%)	492 (20.5%)	378 (18.2%)	386 (19.3%)	**2702 (20.1%)**
Q3	824 (22.6%)	653 (20.0%)	453 (19.8%)	441 (23.4%)	376 (19.5%)	**2747 (21.1%)**
Q4	701 (19.2%)	639 (19.6%)	507 (22.4%)	379 (20.4%)	375 (20.4%)	**2601 (20.3%)**
Q5	602 (16.5%)	567 (17.4%)	371 (17.7%)	340 (18.8%)	368 (20.4%)	**2248 (18.0%)**
**Men ≥ 55**						
Q1	358 (21.1%)	377 (21.7%)	412 (26.1%)	352 (23.5%)	384 (26.0%)	**1883 (23.4%)**
Q2	369 (21.8%)	391 (22.5%)	341 (22.5%)	326 (21.6%)	313 (21.6%)	**1740 (22.1%)**
Q3	380 (22.4%)	358 (20.6%)	294 (20.0%)	343 (23.9%)	280 (19.9%)	**1655 (21.4%)**
Q4	283 (16.7%)	337 (19.4%)	250 (17.4%)	236 (17.3%)	248 (18.0%)	**1354 (17.8%)**
Q5	304 (18.0%)	272 (15.7%)	191 (14.1%)	186 (13.6%)	201 (14.5%)	**1154 (15.4%)**
**Women 16-54**						
Q1	764 (20.3%)	801 (22.4%)	575 (19.9%)	499 (19.1%)	530 (21.7%)	**3169 (20.8%)**
Q2	762 (20.3%)	748 (20.9%)	541 (20.1%)	496 (19.7%)	428 (18.4%)	**2975 (20.0%)**
Q3	815 (21.7%)	714 (20.0%)	485 (18.4%)	520 (22.1%)	415 (18.2%)	**2949 (20.2%)**
Q4	753 (20.1%)	689 (19.3%)	580 (22.6%)	434 (19.2%)	431 (19.7%)	**2887 (20.1%)**
Q5	662 (17.6%)	625 (17.5%)	477 (19.1%)	424 (19.9%)	444 (21.9%)	**2632 (18.9%)**
**Women ≥ 55**						
Q1	401 (19.5%)	417 (21.0%)	495 (26.6%)	415 (23.3%)	437 (25.0%)	**2165 (22.7%)**
Q2	442 (21.5%)	435 (21.9%)	402 (22.0%)	391 (22.5%)	379 (22.3%)	**2049 (22.0%)**
Q3	470 (22.8%)	414 (20.8%)	380 (21.1%)	413 (24.3%)	352 (21.2%)	**2029 (22.0%)**
Q4	355 (17.2%)	372 (18.7%)	301 (17.4%)	262 (16.5%)	286 (17.5%)	**1576 (17.5%)**
Q5	393 (19.1%)	350 (17.6%)	224 (12.9%)	207 (13.4%)	223 (14.0%)	**1397 (15.9%)**

Notes: Respondents with a valid cholesterol measurement (including those taking lipid lowering drugs). Respondent totals are presented unweighted; percentages show distribution across the deprivation fifths (unweighted 1994 and 1998, weighted for non-response from 2003 onwards). Totals may not sum due to rounding. (Q1 = most affluent, Q5 = most deprived).
