# Supplementary material for: Persistent socioeconomic inequalities in cardiovascular risk factors in England over 1994-2008: A time-trend analysis of repeated cross-sectional data
Source: BMC Public Health. 2012 Feb 14;12:129. doi: 10.1186/1471-2458-12-129 (PMC3342910; doi:10.1186/1471-2458-12-129)
Supplement: Additional file 4 — Absolute and relative inequalities in cardiovascular risk factors in men (95% CIs in parentheses) by age-group (using IMD excluding the health domain). The table shows absolute and relative inequalities in cardiovascular risk factors in men calculated using the 'IMD-minus-health domain' quintiles. [file 1471-2458-12-129-S4.PDF]

Additional file 4 Absolute and relative inequalities in cardiovascular risk factors in men (95% CIs in parentheses) by age-group (using IMD excluding the health domain)

	Current smoking	Obesity	Diabetes	High levels of physical activity	High blood pressure (SBP ≥ 140 mmHg)	Raised cholesterol (TC ≥ 5 mmol/l)	≥ 5 portions of fruit & vegetables
**16-54**							
**Absolute difference**							
Model 1a[§]							
Q1 (reference)	0	0	0	0	0	0	0
Q2	2.8 (1.5,4.1)	0.1 (-1.0,1.2)	0.4 (-0.1,1.0)	1.3 (-1.3,3.9)	1.1 (-0.1,2.3)	0.3 (-2.2,2.9)	-2.6 (-4.4,-0.8)
Q3	7.5 (6.1,8.8)	0.6 (-0.4,1.7)	0.5 (-0.1,1.0)	-1.1 (-3.5,1.4)	1.2 (0.0,2.4)	0.0 (-2.6,2.5)	-3.4 (-5.1,-1.6)
Q4	13.2 (11.8,14.6)	2.7 (1.6,3.8)	0.5 (-0.1,1.1)	-0.9 (-3.4,1.6)	0.9 (-0.3,2.2)	-0.8 (-3.6,1.9)	-2.8 (-4.6,-1.0)
Q5	20.2 (18.7,21.6)	1.9 (0.7,3.0)	1.5 (0.8,2.1)	-4.2 (-6.7,-1.7)	2.1 (0.8,3.4)	0.3 (-2.4,2.9)	-5.9 (-7.8,-4.1)
Model 2a[†]	5.0 (4.7,5.4)	0.6 (0.4,0.9)	0.10 (0.06,0.15)	-1.0 (-1.6,-0.5)	-0.3 (-0.4,-0.2)	-0.1 (-0.7,0.5)	-1.2 (-1.6,-0.8)
Model 3a[‡]	p = 0.120[¶]	p = 0.610[¶]	p = 0.023[¶]	p = 0.301[¶]	p = 0.328[¶]	p = 0.662[ǀ]	p = 0.836[¶]
**Relative (PR)**							
Model 1b[§§]							
Q1 (reference)	1	1	1	1	1	1	1
Q2	1.12 (1.06,1.18)	1.01 (0.95,1.08)	1.35 (0.91,2.01)	1.03 (0.95,1.12)	1.07 (0.99,1.16)	0.97 (0.94,1.00)	0.90 (0.83,0.97)
Q3	1.32 (1.25,1.38)	1.04 (0.98,1.11)	1.38 (0.93,2.06)	0.97 (0.89,1.05)	1.08 (1.00,1.17)	0.97 (0.94,1.00)	0.86 (0.80,0.93)
Q4	1.56 (1.49,1.63)	1.17 (1.10,1.25)	1.42 (0.97,2.09)	0.98 (0.90,1.06)	1.06 (0.98,1.15)	0.96 (0.93,0.99)	0.89 (0.82,0.96)
Q5	1.84 (1.76,1.93)	1.12 (1.05,1.19)	2.22 (1.54,3.21)	0.87 (0.80,0.95)	1.15 (1.06,1.25)	0.97 (0.94,1.00)	0.76 (0.70,0.83)
Model 2b[††]	1.17 (1.16,1.18)	1.04 (1.02,1.05)	1.19 (1.09,1.29)	0.97 (0.95,0.99)	1.03 (1.01,1.05)	0.99 (0.98,1.00)	0.95 (0.93,0.96)
Model 3b[‡‡]	p = 0.649[¶]	p = 0.160[¶]	p = 0.179[¶]	p = 0.647[¶]	p = 0.539[¶]	p = 0.676[ǀ]	p = 0.675[¶]
**≥ 55 years**							
**Absolute difference**							
Model 1a[§]							
Q1 (reference)	0	0	0	0	0	0	0
Q2	2.6 (1.5,3.8)	3.2 (1.7,4.7)	-0.4 (-2.1,1.3)	-0.1 (-2.7,2.4)	-0.4 (-2.4,1.7)	0.5 (-2.2,3.2)	0.3 (-2.0,2.6)
Q3	6.6 (5.4,7.9)	3.5 (1.9,5.0)	0.5 (-1.6,2.5)	0.1 (-2.6,2.8)	0.3 (-1.8,2.5)	-2.1 (-5.0,0.9)	-4.0 (-6.3,-1.7)
Q4	11.4 (10.1,12.8)	5.6 (3.9,7.4)	1.0 (-1.3,3.3)	-1.0 (-3.4,1.5)	2.2 (0.0,4.5)	-6.2 (-9.2,-3.1)	-8.3 (-10.7,-5.9)
Q5	18.9 (17.4,20.4)	6.3 (4.5,8.2)	4.1 (2.0,6.1)	-6.6 (-9.3,-3.9)	4.2 (1.8,6.5)	-5.3 (-8.6,-2.1)	-12.7 (-15.1,-10.4)
Model 2a[†]	4.6 (4.3,4.9)	1.5 (1.1,1.9)	0.9 (0.4,1.4)	-1.4 (-1.9,-0.8)	1.1 (0.5,1.6)	-1.7 (-2.4,-1.0)	-3.4 (-3.9,-2.8)
Model 3a[‡]	p = 0.629[¶]	p = 0.064[¶]	p = 0.128[¶]	p = 0.179[¶]	p = 0.239[¶]	p = 0.007[¶]	p = 0.222[¶]
**Relative (PR)**							
Model 1b[§§]							
Q1 (reference)	1	1	1	1	1	1	1
Q2	1.25 (1.13,1.38)	1.16 (1.08,1.24)	0.95 (0.77,1.19)	0.99 (0.86,1.14)	0.99 (0.95,1.04)	1.01 (0.98,1.04)	1.01 (0.94,1.08)
Q3	1.63 (1.48,1.79)	1.17 (1.09,1.25)	1.06 (0.82,1.38)	1.00 (0.86,1.16)	1.01 (0.96,1.06)	0.99 (0.95,1.02)	0.88 (0.81,0.94)
Q4	2.09 (1.91,2.29)	1.27 (1.18,1.37)	1.12 (0.85,1.49)	0.95 (0.82,1.09)	1.05 (1.00,1.10)	0.95 (0.91,0.98)	0.75 (0.68,0.82)
Q5	2.78 (2.55,3.03)	1.31 (1.22,1.41)	1.52 (1.22,1.89)	0.63 (0.52,0.77)	1.09 (1.04,1.15)	0.96 (0.92,1.00)	0.61 (0.55,0.67)
Model 2b[††]	1.30 (1.27,1.32)	1.07 (1.05,1.08)	1.11 (1.05,1.17)	0.92 (0.89,0.95)	1.02 (1.01,1.04)	0.99 (0.99,0.99)	0.89 (0.87,0.91)
Model 3b[‡‡]	p = 0.019[¶]	p = 0.561[¶]	p = 0.462[¶]	p = 0.434[¶]	p = 0.467[¶]	$p \leq 0.001$[¶]	p = 0.056[¶]

PR: Prevalence ratio

[§] *Model 1a:* Percentage point (p.p) difference between IMD quintile and Q1 (adjusted for year and age). Linear regression model: year + age + Q2 + Q3 + Q4 + Q5.

[†] *Model 2a:* p.p difference for unit increase in IMD (fitted as ordinal level variable ranging from 1 to 5). Linear regression model: year + age + IMD. p from the model served as test of linear trend (significance of differences in p.p when moving from one ordinal category to one immediately higher). $p \leq 0.05$ if the 95% CIs do not include 0.

[‡] *Model 3a:* p shown for interaction term testing change in absolute inequality over time. Linear regression model: year + age + IMD + (year × IMD). ([¶] IMD fitted as an ordinal variable; [ǀ]fitted as 4 indicator variables).

[§§] *Model 1b:* PR between IMD quintile and Q1 (adjusted for year and age). Log-binomial regression model: year + age + Q2 + Q3 + Q4 + Q5.

[††] *Model 2b:* PR for unit increase in IMD (fitted as an ordinal level variable). Log-binomial regression model: year + age + IMD. p served as test for linear trend (change in PR when moving from one ordinal category to one immediately higher). $p \leq 0.05$ if the 95% CIs do not include 1.

[‡‡] *Model 3b:* p shown for interaction term testing change in relative inequality over time. Log-binomial regression model: year + age + IMD + (year × IMD). ([¶] IMD fitted as an ordinal variable; [ǀ]IMD fitted as 4 indicator variables).
