# Supplementary material for: Persistent socioeconomic inequalities in cardiovascular risk factors in England over 1994-2008: A time-trend analysis of repeated cross-sectional data
Source: BMC Public Health. 2012 Feb 14;12:129. doi: 10.1186/1471-2458-12-129 (PMC3342910; doi:10.1186/1471-2458-12-129)
Supplement: Additional file 5 — Absolute and relative inequalities in cardiovascular risk factors in women (95% CIs in parentheses) by age-group (using IMD excluding the health domain). The table shows absolute and relative inequalities in cardiovascular risk factors in women calculated using the 'IMD-minus-health domain' quintiles. [file 1471-2458-12-129-S5.PDF]

Additional file 5 Absolute and relative inequalities in cardiovascular risk factors in women (95% CIs in parentheses) by age-group (using IMD excluding the health domain)

	Current smoking	Obesity	Diabetes	High levels of physical activity	High blood pressure (SBP ≥ 140 mmHg)	Raised cholesterol (TC ≥ 5 mmol/l)	≥ 5 portions of fruit & vegetables
**16-54**							
**Absolute difference**							
Model 1a[§]							
Q1 (reference)	0	0	0	0	0	0	0
Q2	3.2 (2.1,4.4)	2.2 (1.2,3.1)	-0.1 (-0.5,0.4)	0.5 (-1.6,2.6)	0.3 (-0.4,1.0)	0.8 (-2.0,3.6)	-1.5 (-3.3,0.3)
Q3	8.0 (6.8,9.2)	4.5 (3.5,5.5)	0.3 (-0.2,0.8)	-0.1 (-2.4,2.1)	1.5 (0.7,2.3)	1.1 (-1.7,3.9)	-3.6 (-5.3,-1.8)
Q4	13.9 (12.7,15.1)	7.0 (6.0,8.1)	0.0 (-0.4,0.4)	-0.7 (-2.8,1.4)	1.7 (0.9,2.5)	0.5 (-2.2,3.2)	-5.6 (-7.3,-3.9)
Q5	20.0 (18.7,21.3)	10.5 (9.4,11.5)	1.2 (0.7,1.8)	-3.3 (-5.4,-1.2)	1.5 (0.7,2.3)	2.2 (-0.6,4.9)	-10.3 (-12.0,-8.5)
Model 2a[†]	5.1 (4.8,5.3)	2.6 (2.3,2.8)	0.3 (0.1,0.4)	-0.8 (-1.2,-0.3)	0.5 (0.3,0.6)	0.4 (-0.2,1.0)	-2.4 (-2.8,-2.0)
Model 3a[‡]	*p* = 0.069[¶]	*p* = 0.582[¶]	*p* = 0.204[¶]	*p* = 0.092[¶]	*p* = 0.015[¶]	*p* = 0.186[ǀ]	*p* = 0.737[¶]
**Relative (PR)**							
Model 1b[§§]							
Q1 (reference)	1	1	1	1	1	1	1
Q2	1.16 (1.10,1.22)	1.16 (1.09,1.24)	0.91 (0.57,1.46)	1.02 (0.94,1.10)	1.04 (0.93,1.15)	1.01 (0.96,1.07)	0.95 (0.90,1.01)
Q3	1.38 (1.32,1.46)	1.33 (1.25,1.42)	1.29 (0.81,2.05)	0.99 (0.91,1.08)	1.22 (1.10,1.35)	1.02 (0.97,1.07)	0.88 (0.83,0.94)
Q4	1.67 (1.60,1.75)	1.53 (1.43,1.63)	1.04 (0.68,1.58)	0.97 (0.90,1.06)	1.24 (1.12,1.37)	1.01 (0.96,1.06)	0.81 (0.77,0.87)
Q5	1.96 (1.87,2.05)	1.78 (1.68,1.89)	2.31 (1.53,3.47)	0.87 (0.80,0.95)	1.22 (1.09,1.36)	1.04 (0.99,1.09)	0.66 (0.62,0.71)
Model 2b[††]	1.19 (1.18,1.20)	1.15 (1.14,1.17)	1.23 (1.12,1.36)	0.97 (0.95,0.99)	1.06 (1.03,1.08)	1.01 (1.00,1.02)	0.91 (0.90,0.93)
Model 3b)[‡‡]	*p* = 0.320[¶]	*p* = 0.032[¶]	*p* = 0.992[¶]	*p* = 0.162[¶]	*p* = 0.001[¶]	*p* = 0.209[ǀ]	*p* = 0.135[¶]
**≥ 55 years**							
**Absolute difference**							
Model 1a[§]							
Q1 (reference)	0	0	0	0	0	0	0
Q2	2.2 (1.1,3.3)	2.8 (1.3,4.3)	-0.3 (-1.5,0.8)	-1.0 (-3.1,1.0)	0.9 (-1.0,2.8)	-0.3 (-2.3,1.6)	-2.3 (-4.4,-0.2)
Q3	5.3 (4.1,6.5)	4.2 (2.6,5.7)	0.0 (-1.1,1.2)	0.1 (-2.2,2.3)	1.3 (-0.7,3.2)	-0.8 (-2.8,1.2)	-5.5 (-7.7,-3.3)
Q4	9.8 (8.5,11.1)	8.0 (6.3,9.7)	2.6 (1.1,4.2)	-2.8 (-4.9,-0.6)	2.4 (0.3,4.5)	-4.5 (-7.1,-1.9)	-9.0 (-11.3,-6.8)
Q5	17.3 (15.8,18.8)	9.0 (7.3,10.8)	4.7 (3.1,6.2)	-5.2 (-7.4,-2.9)	3.7 (1.6,5.9)	-4.3 (-6.8,-1.7)	-15.5 (-17.7,-13.3)
Model 2a[†]	4.2 (3.9,4.5)	2.3 (1.9,2.7)	1.2 (0.9,1.6)	-1.2 (-1.7,-0.7)	0.9 (0.4,1.4)	-1.3 (-1.9,-0.7)	-3.7 (-4.2,-3.2)
Mode13a[‡]	*p* = 0.536[¶]	*p* = 0.060[¶]	*p* = 0.005[¶]	*p* = 0.064[¶]	*p* = 0.452[¶]	*p* ≤ 0.001[¶]	*p* = 0.389[¶]
**Relative (PR)**							
Model 1b[§§]							
Q1 (reference)	1	1	1	1	1	1	1
Q2	1.18 (1.08,1.29)	1.12 (1.05,1.19)	0.93 (0.72,1.19)	0.94 (0.83,1.07)	1.02 (0.98,1.06)	1.00 (0.98,1.02)	0.94 (0.88,1.00)
Q3	1.45 (1.33,1.58)	1.18 (1.11,1.25)	1.01 (0.79,1.29)	1.01 (0.88,1.15)	1.03 (0.98,1.07)	1.01 (0.99,1.03)	0.85 (0.80,0.91)
Q4	1.84 (1.69,2.00)	1.34 (1.26,1.43)	1.56 (1.21,2.01)	0.84 (0.73,0.96)	1.05 (1.01,1.10)	0.97 (0.94,0.99)	0.75 (0.70,0.81)
Q5	2.49 (2.30,2.69)	1.39 (1.31,1.48)	2.02 (1.61,2.53)	0.69 (0.58,0.82)	1.08 (1.03,1.13)	0.97 (0.95,1.00)	0.57 (0.52,0.63)
Model 2b[††]	1.26 (1.24,1.29)	1.09 (1.07,1.10)	1.23 (1.16,1.30)	0.92 (0.89,0.96)	1.02 (1.01,1.03)	0.99 (0.99,1.00)	0.88 (0.87,0.90)
Model 3b[‡‡]	*p* = 0.059[¶]	*p* = 0.353[¶]	*p* = 0.444[¶]	*p* = 0.292[¶]	*p* = 0.162[¶ ǀ]	*p* ≤ 0.001[¶]	*p* = 0.043[¶]

PR: Prevalence ratio

[§] *Model 1a:* Percentage point (p.p) difference between IMD quintile and Q1 (adjusted for year and age). Linear regression model: year + age + Q2 + Q3 + Q4 + Q5.

[†] *Model 2a:* p.p difference for unit increase in IMD (fitted as ordinal level variable ranging from 1 to 5). Linear regression model: year + age + IMD. *p* from the model served as test of linear trend (significance of differences in p.p when moving from one ordinal category to one immediately higher). $p \leq 0.05$ if the 95% CIs do not include 0.

[‡] *Model 3a: p* shown for interaction term testing change in absolute inequality over time. Linear regression model: year + age + IMD + (year × IMD). ([¶] IMD fitted as an ordinal variable; [ǀ]fitted as 4 indicator variables).

[§§] *Model 1b:* PR between IMD quintile and Q1 (adjusted for year and age). Log-binomial regression model: year + age + Q2 + Q3 + Q4 + Q5.

[††] *Model 2b:* PR for unit increase in IMD (fitted as an ordinal level variable). Log-binomial regression model: year + age + IMD. *p* served as test for linear trend (change in PR when moving from one ordinal category to one immediately higher). $p \leq 0.05$ if the 95% CIs do not include 1.

[‡‡] *Model 3b: p* shown for interaction term testing change in relative inequality over time. Log-binomial regression model: year + age + IMD + (year × IMD). ([¶] IMD fitted as an ordinal variable; [ǀ]IMD fitted as 4 indicator variables).

∫ Model fitted using Poisson regression due to log-binomial regression failing to converge.
