# Supplementary material for: Persistent socioeconomic inequalities in cardiovascular risk factors in England over 1994-2008: A time-trend analysis of repeated cross-sectional data
Source: BMC Public Health. 2012 Feb 14;12:129. doi: 10.1186/1471-2458-12-129 (PMC3342910; doi:10.1186/1471-2458-12-129)
Supplement: Additional file 6 — Absolute change in cardiovascular risk factors in men aged 16-54 years, by deprivation quintiles. The table shows the absolute change in cardiovascular risk factors between the most affluent and most deprived fifths over 1994-2008 with accompanying 95% confidence intervals. [file 1471-2458-12-129-S6.PDF]

ADDITIONAL FILE 6 Absolute change in cardiovascular risk factors in men aged 16-54 years, by deprivation quintiles (1/2)

		England	Q1 Most affluent	Q2	Q3	Q4	Q5 Most deprived
**Cigarette Smoking (%)**	1994	**32.7** (31.4 to 34.1)	26.4 (23.4 to 29.4)	28.4 (25.3 to 31.4)	30.6 (27.7 to 33.5)	38.2 (34.7 to 41.8)	42.2 (38.9 to 45.5)
	2008	**28.2** (26.6 to 29.8)	21.4 (17.8 to 25.1)	23.0 (19.7 to 26.4)	24.7 (21.6 to 27.9)	32.3 (28.6 to 35.9)	40.6 (36.7 to 44.5)
	Change from 1994	***-4.6** (-6.7 to -2.4)*	*-5.0 (-9.7 to -0.3)*	*-5.3 (-9.9 to -0.8)*	*-5.9 (-10.1 to -1.6)*	*-5.9 (-11.0 to -0.8)*	*-1.6 (-6.7 to 3.5)*
	P-value	***(<0.000)***	*(0.039)*	*(0.021)*	*(0.007)*	*(0.022)*	*(0.539)*
**BMI (kg/m^2)**	1994	**25.5** (25.4 to 25.7)	25.5 (25.3 to 25.8)	25.6 (25.3 to 25.8)	25.5 (25.3 to 25.8)	25.6 (25.3 to 25.9)	25.4 (25.0 to 25.7)
	2008	**26.5** (26.3 to 26.7)	26.5 (26.1 to 26.9)	26.8 (26.4 to 27.1)	26.4 (26.0 to 26.8)	26.5 (26.1 to 26.9)	26.5 (26.1 to 26.9)
	Change from 1994	***1.0** (0.8 to 1.2)*	*1.0 (0.5 to 1.4)*	*1.2 (0.7 to 1.6)*	*0.8 (0.4 to 1.3)*	*0.9 (0.4 to 1.4)*	*1.2 (0.7 to 1.7)*
	P-value	***(<0.000)***	*(<0.000)*	*(<0.000)*	*(<0.000)*	*(<0.000)*	*(<0.000)*
**Obesity (%)**	1994	**12.1** (11.1 to 13.0)	11.4 (9.4 to 13.4)	9.9 (8.0 to 11.9)	12.8 (10.8 to 14.7)	13.8 (11.4 to 16.2)	12.5 (10.1 to 14.9)
	2008	**20.5** (19.1 to 21.9)	20.4 (17.3 to 23.6)	21.3 (18.1 to 24.5)	18.4 (15.3 to 21.4)	21.3 (17.9 to 24.6)	21.2 (18.1 to 24.2)
	Change from 1994	***8.4** (6.7 to 10.2)*	*9.0 (5.3 to 12.7)*	*11.4 (7.6 to 15.1)*	*5.6 (2.0 to 9.3)*	*7.4 (3.3 to 11.5)*	*8.7 (4.8 to 12.5)*
	P-value	***(<0.000)***	*(<0.000)*	*(<0.000)*	*(0.002)*	*(<0.000)*	*(<0.000)*
**Diabetes (%)**	1994	**1.3** (1.0 to 1.6)	1.3 (0.6 to 2.0)	1.4 (0.6 to 2.1)	0.9 (0.3 to 1.5)	0.8 (0.2 to 1.5)	2.3 (1.2 to 3.3)
	2006	**2.6** (2.1 to 3.2)	1.5 (0.6 to 2.4)	2.8 (1.4 to 4.2)	2.2 (1.2 to 3.2)	2.8 (1.5 to 4.0)	4.1 (2.7 to 5.6)
	Change from 1994	***1.3** (0.7 to 2.0)*	*0.2 (-0.9 to 1.4)*	*1.4 (-0.1 to 3.0)*	*1.3 (0.1 to 2.5)*	*1.9 (0.5 to 3.3)*	*1.9 (0.1 to 3.6)*
	P-value	***(<0.000)***	*(0.667)*	*(0.069)*	*(0.027)*	*(0.007)*	*(0.036)*
**High physical activity (%)**	1998	**27.8** (26.4 to 29.3)	28.2 (25.2 to 31.1)	29.5 (26.5 to 32.5)	28.3 (25.2 to 31.5)	26.8 (23.9 to 29.7)	26.2 (23.0 to 29.3)
	2008	**36.3** (34.6 to 38.0)	38.7 (34.7 to 42.7)	36.5 (32.3 to 40.6)	36.9 (33.2 to 40.5)	36.8 (33.1 to 40.4)	32.4 (28.8 to 36.1)
	Change from 1998	***8.5** (6.3 to 10.7)*	*10.6 (5.6 to 15.6)*	*7.0 (1.9 to 12.1)*	*8.5 (3.7 to 13.3)*	*10.0 (5.3 to 14.6)*	*6.3 (1.5 to 11.1)*
	P-value	***(<0.000)***	*(<0.000)*	*(0.007)*	*(0.001)*	*(<0.000)*	*(0.010)*
**SBP (mmHg)**	1994	**129.5** (129.0 to 129.9)	129.3 (128.3 to 130.2)	129.1 (128.1 to 130.1)	129.6 (128.7 to 130.5)	129.2 (128.3 to 130.1)	130.3 (129.3 to 131.3)
	2008	**126.2** (125.6 to 126.7)	124.5 (123.4 to 125.7)	126.8 (125.5 to 128.1)	127.3 (126.0 to 128.6)	126.3 (125.1 to 127.6)	126.0 (124.6 to 127.4)
	Change from 1994	***-3.3** (-4.0 to -2.6)*	*-4.7 (-6.2 to -3.2)*	*-2.3 (-3.9 to -0.7)*	*-2.3 (-3.8 to -0.7)*	*-2.9 (-4.4 to -1.3)*	*-4.3 (-6.0 to -2.6)*
	P-value	***(<0.000)***	*(<0.000)*	*(0.005)*	*(0.005)*	*(<0.000)*	*(<0.000)*
**SBP ≥ 140 mmHg (%)**	1994	**17.0** (15.6 to 18.3)	15.8 (13.1 to 18.4)	17.7 (15.0 to 20.5)	17.2 (14.5 to 20.0)	15.1 (12.4 to 17.8)	19.3 (16.0 to 22.5)
	2008	**13.5** (12.0 to 15.0)	10.0 (7.2 to 12.7)	13.9 (10.4 to 17.3)	16.1 (12.4 to 19.8)	14.2 (11.0 to 17.4)	13.8 (10.3 to 17.4)
	Change from 1994	***-3.5** (-5.5 to -1.4)*	*-5.8 (-9.6 to -1.9)*	*-3.9 (-8.3 to 0.6)*	*-1.1 (-5.7 to 3.5)*	*-0.9 (-5.0 to 3.3)*	*-5.4 (-10.2 to -0.6)*
	P-value	***(0.001)***	*(0.003)*	*(0.086)*	*(0.636)*	*(0.678)*	*(0.027)*

ADDITIONAL FILE 6 Absolute change in cardiovascular risk factors in men aged 16-54 years, by deprivation quintiles (2/2)

		England	Q1<br>Most affluent	Q2	Q3	Q4	Q5<br>Most deprived
**Total cholesterol (mmol/l)**	1994	**5.6** (5.5 to 5.6)	5.5 (5.5 to 5.6)	5.6 (5.5 to 5.7)	5.5 (5.4 to 5.6)	5.5 (5.4 to 5.6)	5.6 (5.5 to 5.7)
	2008	**5.2** (5.2 to 5.3)	5.2 (5.1 to 5.3)	5.2 (5.1 to 5.4)	5.2 (5.0 to 5.3)	5.3 (5.1 to 5.4)	5.2 (5.1 to 5.3)
	Change from 1994	***-0.3*** *(-0.4 to -0.3)*	*-0.4 (-0.5 to -0.2)*	*-0.4 (-0.5 to -0.2)*	*-0.4 (-0.5 to -0.2)*	*-0.2 (-0.4 to 0.0)*	*-0.4 (-0.6 to -0.2)*
	P-value	***(<0.000)***	*(<0.000)*	*(<0.000)*	*(<0.000)*	*(0.016)*	*(<0.000)*
**Total cholesterol ≥ 5.0 mmol/l (%)**	1994	**66.1** (64.3 to 67.8)	67.9 (64.5 to 71.3)	65.7 (61.9 to 69.5)	65.3 (61.4 to 69.2)	64.8 (61.0 to 68.6)	66.6 (62.5 to 70.7)
	2008	**56.3** (53.6 to 59.1)	55.4 (49.8 to 61.0)	56.1 (50.3 to 61.9)	56.0 (50.5 to 61.6)	56.1 (49.7 to 62.6)	58.3 (52.8 to 63.8)
	Change from 1994	**-9.7** (-12.9 to -6.5)	-12.5 (-19.0 to -6.0)	-9.6 (-16.5 to -2.7)	-9.2 (-16.0 to -2.4)	-8.7 (-16.1 to -1.2)	-8.3 (-15.2 to -1.5)
	P-value	***(<0.000)***	*(<0.000)*	*(0.006)*	*(0.008)*	*(0.023)*	*(0.017)*
**Fruit and vegetable consumption (portions per day)**	2001	**3.0** (3.0 to 3.1)	3.3 (3.1 to 3.4)	3.1 (3.0 to 3.3)	3.1 (2.9 to 3.2)	2.8 (2.7 to 3.0)	2.9 (2.7 to 3.0)
	2008	**3.4** (3.3 to 3.4)	3.5 (3.4 to 3.7)	3.5 (3.3 to 3.7)	3.3 (3.1 to 3.5)	3.4 (3.2 to 3.6)	3.0 (2.8 to 3.2)
	Change from 2001	***0.3*** *(0.2 to 0.4)*	*0.3 (0.0 to 0.5)*	*0.4 (0.1 to 0.6)*	*0.3 (0.0 to 0.5)*	*0.5 (0.2 to 0.8)*	*0.2 (-0.1 to 0.4)*
	P-value	***(<0.000)***	*(0.040)*	*(0.003)*	*(0.058)*	*(<0.000)*	*(0.234)*
**Fruit and vegetable consumption (≥ 5 portions per day) (%)**	2001	**19.4** (18.2 to 20.7)	21.7 (19.1 to 24.4)	19.7 (17.1 to 22.3)	19.5 (16.6 to 22.4)	17.3 (14.8 to 19.7)	18.8 (15.9 to 21.7)
	2008	**23.3** (21.8 to 24.8)	26.4 (23.0 to 29.8)	23.9 (20.6 to 27.2)	23.0 (19.5 to 26.4)	23.0 (19.9 to 26.2)	19.8 (16.5 to 23.1)
	Change from 2001	**3.9** (1.9 to 5.8)	4.7 (0.3 to 9.0)	4.2 (0.0 to 8.4)	3.5 (-1.0 to 7.9)	5.8 (1.8 to 9.7)	1.0 (-3.4 to 5.4)
	P-value	***(<0.000)***	*(0.035)*	*(0.052)*	*(0.130)*	*(0.004)*	*(0.649)*
