# Supplementary material for: Persistent socioeconomic inequalities in cardiovascular risk factors in England over 1994-2008: A time-trend analysis of repeated cross-sectional data
Source: BMC Public Health. 2012 Feb 14;12:129. doi: 10.1186/1471-2458-12-129 (PMC3342910; doi:10.1186/1471-2458-12-129)
Supplement: Additional file 7 — Absolute change in cardiovascular risk factors in men aged ≥ 55 years, by deprivation quintiles. The table shows the absolute change in cardiovascular risk factors between the most affluent and most deprived fifths over 1994-2008 with accompanying 95% confidence intervals. [file 1471-2458-12-129-S7.PDF]

ADDITIONAL FILE 7 Absolute change in cardiovascular risk factors in men aged ≥ 55 years, by deprivation quintiles (1/2)

		**England**	**Q1 Most affluent**	**Q2**	**Q3**	**Q4**	**Q5 Most deprived**
**Cigarette Smoking (%)**	1994	**19.9** (18.3 to 21.6)	12.1 (9.3 to 14.9)	15.5 (12.3 to 18.6)	18.8 (15.3 to 22.3)	22.1 (18.2 to 26.1)	33.4 (28.7 to 38.0)
	2008	**15.0** (13.5 to 16.4)	6.5 (4.6 to 8.4)	11.1 (8.4 to 13.7)	11.4 (8.5 to 14.3)	19.8 (15.9 to 23.7)	27.5 (23.3 to 31.7)
	Change from 1994	***-4.9** (-7.1 to-2.7)*	*-5.6 (-9.0 to -2.2)*	*-4.4 (-8.5 to -0.3)*	*-7.4 (-12.0 to -2.9)*	*-2.3 (-7.9 to 3.2)*	*-5.8 (-12.1 to 0.4)*
	P-value	***(<0.000)***	*(0.001)*	*(0.034)*	*(0.001)*	*(0.411)*	*(0.067)*
**BMI (kg/m^2)**	1994	**26.9** (26.8 to 27.1)	26.8 (26.4 to 27.2)	26.9 (26.5 to 27.2)	26.9 (26.6 to 27.2)	27.2 (26.8 to 27.6)	26.9 (26.5 to 27.3)
	2008	**28.4** (28.2 to 28.6)	28.1 (27.7 to 28.4)	28.7 (28.3 to 29.1)	28.5 (28.1 to 28.9)	28.3 (27.8 to 28.8)	28.4 (27.9 to 29.0)
	Change from 1994	***1.5** (1.2 to 1.7)*	*1.3 (0.8 to 1.8)*	*1.9 (1.3 to 2.4)*	*1.6 (1.1 to 2.1)*	*1.1 (0.4 to 1.7)*	*1.5 (0.8 to 2.2)*
	P-value	***(<0.000)***	*(<0.000)*	*(<0.000)*	*(<0.000)*	*(0.001)*	*(<0.000)*
**Obesity (%)**	1994	**17.3** (15.7 to 18.8)	15.4 (11.8 to 18.9)	15.6 (12.2 to 19.0)	16.7 (13.3 to 20.0)	19.8 (15.9 to 23.7)	19.5 (15.4 to 23.6)
	2008	**31.8** (29.8 to 33.8)	27.5 (23.8 to 31.1)	32.9 (28.6 to 37.1)	32.5 (28.3 to 36.7)	32.2 (27.2 to 37.1)	34.4 (29.0 to 39.8)
	Change from 1994	***14.5** (12.0 to 17.0)*	*12.1 (7.0 to 17.2)*	*17.3 (11.9 to 22.7)*	*15.9 (10.5 to 21.2)*	*12.4 (6.1 to 18.6)*	*14.9 (8.1 to 21.7)*
	P-value	***(<0.000)***	*(<0.000)*	*(<0.000)*	*(<0.000)*	*(<0.000)*	*(<0.000)*
**Diabetes (%)**	1994	**6.4** (5.4 to 7.4)	7.0 (5.0 to 9.1)	4.5 (2.6 to 6.3)	5.4 (3.4 to 7.3)	7.6 (5.1 to 10.1)	8.0 (5.2 to 10.8)
	2006	**12.0** (10.3 to 13.6)	10.2 (7.1 to 13.2)	10.6 (7.3 to 13.8)	10.9 (8.1 to 13.7)	11.3 (7.5 to 15.2)	17.4 (12.4 to 22.4)
	Change from 1994	***5.5** (3.6 to 7.4)*	*3.1 (-0.6 to 6.8)*	*6.1 (2.4 to 9.8)*	*5.6 (2.1 to 9.0)*	*3.7 (-0.8 to 8.3)*	*9.4 (3.7 to 15.1)*
	P-value	***(<0.000)***	*(0.100)*	*(0.001)*	*(0.002)*	*(0.110)*	*(0.001)*
**High physical activity (%)**	1998	**13.7** (12.3 to 15.2)	15.3 (12.0 to 18.6)	15.7 (12.3 to 19.0)	13.1 (9.8 to 16.3)	14.3 (11.2 to 17.4)	9.8 (6.9 to 12.8)
	2008	**17.9** (16.4 to 19.5)	21.0 (17.9 to 24.1)	20.7 (16.9 to 24.6)	19.1 (15.9 to 22.4)	16.2 (12.3 to 20.1)	12.1 (9.0 to 15.3)
	Change from 1998	***4.2** (2.1 to 6.3)*	*5.7 (1.2 to 10.2)*	*5.1 (0.0 to 10.2)*	*6.0 (1.5 to 10.6)*	*1.9 (-3.1 to 6.9)*	*2.3 (-2.0 to 6.6)*
	P-value	***(<0.000)***	*(0.013)*	*(0.052)*	*(0.010)*	*(0.454)*	*(0.298)*
**SBP (mmHg)**	1994	**143.1** (142.2 to 143.9)	143.0 (141.2 to 144.8)	142.2 (140.5 to 143.9)	142.5 (140.7 to 144.2)	143.0 (141.1 to 144.9)	145.1 (143.1 to 147.2)
	2008	**135.5** (134.7 to 136.4)	135.1 (133.5 to 136.6)	135.5 (133.7 to 137.3)	135.3 (133.5 to 137.2)	135.9 (133.8 to 138.0)	136.1 (133.8 to 138.4)
	Change from 1994	***-7.5** (-8.7 to -6.3)*	*-7.9 (-10.3 to -5.5)*	*-6.7 (-9.2 to -4.3)*	*-7.1 (-9.7 to -4.6)*	*-7.1 (-10.0 to -4.3)*	*-9.0 (-12.1 to -5.9)*
	P-value	***(<0.000)***	*(<0.000)*	*(<0.000)*	*(<0.000)*	*(<0.000)*	*(<0.000)*
**SBP ≥ 140 mmHg (%)**	1994	**51.8** (49.4 to 54.1)	52.9 (48.2 to 57.6)	48.9 (44.1 to 53.7)	48.6 (43.6 to 53.6)	53.4 (48.0 to 58.7)	56.2 (50.9 to 61.6)
	2008	**37.4** (35.1 to 39.7)	37.0 (32.4 to 41.5)	39.5 (34.5 to 44.5)	35.4 (30.5 to 40.4)	37.0 (31.3 to 42.7)	38.1 (31.9 to 44.4)
	Change from 1994	-14.4 (-17.7 to -11.1)	-15.9 (-22.5 to -9.4)	-9.4 (-16.3 to -2.4)	-13.1 (-20.2 to -6.1)	-16.4 (-24.2 to -8.5)	-18.1 (-26.4 to -9.8)
	P-value	***(<0.000)***	*(<0.000)*	*(0.008)*	*(<0.000)*	*(<0.000)*	*(<0.000)*

ADDITIONAL FILE 7 Absolute change in cardiovascular risk factors in men aged ≥ 55 years, by deprivation quintiles (2/2)

		England	Q1 Most affluent	Q2	Q3	Q4	Q5 Most deprived
**Total cholesterol (mmol/l)**	1994	**6.2** (6.1 to 6.2)	6.3 (6.2 to 6.4)	6.1 (6.0 to 6.2)	6.1 (6.0 to 6.2)	6.1 (6.0 to 6.3)	6.2 (6.1 to 6.3)
	2008	**5.3** (5.2 to 5.3)	5.4 (5.3 to 5.5)	5.3 (5.1 to 5.4)	5.2 (5.1 to 5.4)	5.2 (5.1 to 5.4)	5.1 (4.9 to 5.2)
	Change from 1994	***-0.9*** *(-1.0 to -0.8)*	*-0.9 (-1.0 to -0.7)*	*-0.8 (-1.0 to -0.6)*	*-0.9 (-1.1 to -0.7)*	*-0.9 (-1.2 to -0.7)*	*-1.1 (-1.3 to -0.9)*
	P-value	***(<0.000)***	*(<0.000)*	*(<0.000)*	*(<0.000)*	*(<0.000)*	*(<0.000)*
**Total cholesterol ≥ 5.0 mmol/l (%)**	1994	**87.2** (85.6 to 88.8)	89.6 (86.4 to 92.8)	87.6 (84.2 to 91.1)	86.2 (82.5 to 89.9)	85.7 (81.7 to 89.7)	86.6 (82.9 to 90.4)
	2008	**58.1** (55.3 to 60.8)	67.2 (62.5 to 71.9)	61.3 (55.3 to 67.2)	54.6 (48.4 to 60.7)	53.2 (46.9 to 59.5)	52.5 (45.3 to 59.8)
	Change from 1994	**-29.1** (-32.3 to -25.9)	-22.4 (-28.1 to -16.7)	-26.4 (-33.3 to -19.5)	-31.7 (-38.9 to -24.5)	-32.5 (-40.0 to -25.0)	-34.1 (-42.2 to -25.9)
	P-value	***(<0.000)***	*(<0.000)*	*(<0.000)*	*(<0.000)*	*(<0.000)*	*(<0.000)*
**Fruit and vegetable consumption (portions per day)**	2001	**3.6** (3.5 to 3.7)	4.0 (3.8 to 4.2)	3.9 (3.7 to 4.1)	3.7 (3.4 to 3.9)	3.4 (3.2 to 3.7)	2.9 (2.7 to 3.1)
	2008	**3.9** (3.8 to 4.0)	4.1 (3.9 to 4.3)	4.1 (4.0 to 4.3)	4.1 (3.8 to 4.3)	3.6 (3.4 to 3.8)	3.4 (3.1 to 3.7)
	Change from 2001	***0.3*** *(0.1 to 0.4)*	*0.1 (-0.2 to 0.4)*	*0.2 (-0.1 to 0.5)*	*0.4 (0.1 to 0.7)*	*0.2 (-0.1 to 0.5)*	*0.6 (0.2 to 0.9)*
	P-value	***(<0.000)***	*(0.448)*	*(0.134)*	*(0.020)*	*(0.242)*	*(0.002)*
**Fruit and vegetable consumption (≥ 5 portions per day) (%)**	2001	**26.2** (24.4 to 28.0)	30.4 (26.6 to 34.2)	32.5 (28.5 to 36.4)	27.3 (23.3 to 31.3)	23.4 (19.3 to 27.5)	15.9 (12.1 to 19.6)
	2008	**29.0** (27.2 to 30.9)	31.3 (27.5 to 35.1)	33.2 (29.4 to 37.0)	31.7 (27.5 to 35.9)	24.6 (20.7 to 28.5)	24.0 (19.6 to 28.3)
	Change from 2001	**2.8** (0.2 to 5.4)	0.9 (-4.4 to 6.3)	0.7 (-4.8 to 6.2)	4.4 (-1.3 to 10.2)	1.2 (-4.5 to 6.8)	8.1 (2.3 to 13.9)
	P-value	***(0.032)***	*(0.733)*	*(0.800)*	*(0.132)*	*(0.679)*	*(0.006)*
