# Supplementary material for: Persistent socioeconomic inequalities in cardiovascular risk factors in England over 1994-2008: A time-trend analysis of repeated cross-sectional data
Source: BMC Public Health. 2012 Feb 14;12:129. doi: 10.1186/1471-2458-12-129 (PMC3342910; doi:10.1186/1471-2458-12-129)
Supplement: Additional file 8 — Absolute change in cardiovascular risk factors in women aged 16-54 years, by deprivation quintiles. The table shows the absolute change in cardiovascular risk factors between the most affluent and most deprived fifths over 1994-2008 with accompanying 95% confidence intervals. [file 1471-2458-12-129-S8.PDF]

ADDITIONAL FILE 8 Absolute change in cardiovascular risk factors in women aged 16-54 years, by deprivation quintiles (1/2)

		England	Q1<br>Most affluent	Q2	Q3	Q4	Q5<br>Most deprived
**Cigarette Smoking (%)**	1994	**31.1** (29.7 to 32.4)	21.4 (18.8 to 24.0)	24.9 (22.0 to 27.8)	30.0 (27.1 to 32.9)	37.0 (34.2 to 39.9)	42.5 (39.3 to 45.7)
	2008	**23.6** (22.3 to 25.0)	15.9 (13.3 to 18.4)	18.4 (15.6 to 21.2)	22.3 (19.4 to 25.2)	29.3 (26.2 to 32.4)	33.2 (29.9 to 36.4)
	Change from 1994	***-7.5*** *(-9.4 to -5.6)*	*-5.5 (-9.2 to -1.9)*	*-6.5 (-10.5 to -2.5)*	*-7.7 (-11.8 to -3.6)*	*-7.7 (-12.0 to -3.5)*	*-9.3 (-13.9 to -4.8)*
	P-value	***(<0.000)***	*(0.003)*	*(0.001)*	*(<0.000)*	*(<0.000)*	*(<0.000)*
**BMI (kg/m^2)**	1994	**25.1** (24.9 to 25.2)	24.5 (24.3 to 24.8)	24.7 (24.4 to 25.0)	25.0 (24.7 to 25.3)	25.3 (25.0 to 25.7)	25.9 (25.5 to 26.3)
	2008	**26.2** (26.0 to 26.4)	25.8 (25.4 to 26.2)	25.5 (25.2 to 25.9)	26.1 (25.7 to 26.4)	26.7 (26.3 to 27.2)	27.2 (26.8 to 27.6)
	Change from 1994	***1.2*** *(0.9 to 1.4)*	*1.2 (0.8 to 1.7)*	*0.8 (0.4 to 1.3)*	*1.1 (0.6 to 1.6)*	*1.4 (0.8 to 2.0)*	*1.3 (0.7 to 1.9)*
	P-value	***(<0.000)***	*(<0.000)*	*(<0.000)*	*(<0.000)*	*(<0.000)*	*(<0.000)*
**Obesity (%)**	1994	**14.0** (13.0 to 15.0)	9.3 (7.6 to 10.9)	11.0 (9.1 to 12.9)	14.9 (12.6 to 17.2)	15.8 (13.3 to 18.2)	19.3 (16.5 to 22.0)
	2008	**21.6** (20.3 to 22.9)	18.1 (15.3 to 20.9)	17.0 (14.4 to 19.6)	19.0 (16.2 to 21.8)	25.5 (22.4 to 28.6)	29.2 (26.0 to 32.3)
	Change from 1994	***7.6*** *(6.0 to 9.3)*	*8.8 (5.6 to 12.1)*	*6.0 (2.8 to 9.2)*	*4.1 (0.5 to 7.7)*	*9.8 (5.8 to 13.7)*	*9.9 (5.7 to 14.1)*
	P-value	***(<0.000)***	*(<0.000)*	*(<0.000)*	*(0.027)*	*(<0.000)*	*(<0.000)*
**Diabetes (%)**	1994	**0.8** (0.6 to 1.0)	0.8 (0.3 to 1.4)	0.8 (0.3 to 1.2)	1.0 (0.4 to 1.6)	0.3 (0.0 to 0.6)	1.2 (0.5 to 1.8)
	2006	**1.8** (1.4 to 2.2)	1.4 (0.6 to 2.3)	1.3 (0.5 to 2.0)	1.9 (1.0 to 2.8)	1.6 (0.7 to 2.4)	2.7 (1.7 to 3.8)
	Change from 1994	***1.0*** *(0.5 to 1.4)*	*0.6 (-0.4 to 1.6)*	*0.5 (-0.4 to 1.4)*	*0.9 (-0.1 to 2.0)*	*1.3 (0.4 to 2.2)*	*1.6 (0.3 to 2.9)*
	P-value	***(<0.000)***	*(0.250)*	*(0.248)*	*(0.085)*	*(0.005)*	*(0.014)*
**High physical activity (%)**	1998	**23.4** (22.1 to 24.6)	23.1 (20.6 to 25.6)	25.0 (22.1 to 27.9)	23.6 (20.9 to 26.3)	23.4 (20.8 to 26.0)	21.6 (19.1 to 24.0)
	2008	**30.5** (29.1 to 32.0)	33.5 (30.2 to 36.9)	32.5 (29.2 to 35.8)	29.4 (26.2 to 32.5)	29.5 (26.4 to 32.6)	27.4 (24.4 to 30.5)
	Change from 1998	***7.2*** *(5.3 to 9.1)*	*10.4 (6.2 to 14.6)*	*7.5 (3.1 to 11.9)*	*5.8 (1.6 to 9.0)*	*6.1 (2.0 to 10.1)*	*5.9 (2.0 to 9.8)*
	P-value	***(<0.000)***	*(<0.000)*	*(0.001)*	*(0.007)*	*(0.003)*	*(0.003)*
**SBP (mmHg)**	1994	**122.6** (122.2 to 123.0)	122.8 (122.0 to 123.6)	122.0 (121.1 to 122.9)	122.9 (121.9 to 123.8)	122.3 (121.4 to 123.3)	123.0 (122.1 to 123.9)
	2008	**116.4** (115.8 to 117.0)	115.7 (114.5 to 116.9)	116.0 (114.7 to 117.2)	116.6 (115.2 to 117.9)	116.7 (115.5 to 117.9)	117.2 (115.9 to 118.5)
	Change from 1994	***-6.2*** *(-6.9 to -5.5)*	*-7.1 (-8.5 to -5.6)*	*-6.0 (-7.5 to -4.6)*	*-6.3 (-7.9 to -4.6)*	*-5.7 (-7.2 to -4.2)*	*-5.8 (-7.3 to -4.2)*
	P-value	***(<0.000)***	*(<0.000)*	*(<0.000)*	*(<0.000)*	*(<0.000)*	*(<0.000)*
**SBP ≥ 140 mmHg (%)**	1994	**8.7** (7.8 to 9.6)	8.7 (7.1 to 10.3)	8.3 (6.4 to 10.2)	9.1 (7.1 to 11.0)	8.5 (6.7 to 10.3)	8.9 (7.0 to 10.9)
	2008	**5.5** (4.6 to 6.3)	4.7 (3.2 to 6.1)	3.0 (1.7 to 4.3)	6.2 (4.2 to 8.3)	7.3 (5.1 to 9.4)	6.4 (4.3 to 8.5)
	Change from 1994	***-3.2*** *(-4.4 to -2.1)*	*-4.0 (-6.2 to -1.9)*	*-5.3 (-7.6 to -3.0)*	*-2.8 (-5.6 to 0.0)*	*-1.2 (-4.0 to 1.6)*	*-2.6 (-5.4 to 0.3)*
	P-value	***(<0.000)***	*(<0.000)*	*(<0.000)*	*(0.048)*	*(0.406)*	*(0.081)*

ADDITIONAL FILE 8 Absolute change in cardiovascular risk factors in women aged 16-54 years, by deprivation quintiles (2/2)

		England	Q1 Most affluent	Q2	Q3	Q4	Q5 Most deprived
**Total cholesterol (mmol/l)**	1994	**5.4** (5.4 to 5.4)	5.3 (5.2 to 5.4)	5.4 (5.3 to 5.5)	5.4 (5.4 to 5.5)	5.4 (5.3 to 5.5)	5.4 (5.4 to 5.5)
	2008	**5.1** (5.0 to 5.1)	5.0 (4.9 to 5.1)	5.1 (5.0 to 5.2)	5.1 (5.0 to 5.3)	5.0 (4.9 to 5.1)	5.1 (5.0 to 5.2)
	Change from 1994	***-0.3** (-0.4 to -0.3)*	*-0.3 (-0.4 to -0.2)*	*-0.2(-0.4 to -0.1)*	*-0.3 (-0.5 to -0.2)*	*-0.4 (-0.5 to -0.3)*	*-0.3 (-0.5 to -0.2)*
	P-value	***(<0.000)***	*(<0.000)*	*(0.001)*	*(<0.000)*	*(<0.000)*	*(<0.000)*
**Total cholesterol ≥ 5.0 mmol/l (%)**	1994	**64.3** (62.6 to 66.1)	62.5 (58.2 to 66.7)	63.8 (59.9 to 67.7)	67.7 (64.3 to 71.1)	62.9 (59.1 to 66.8)	64.4 (60.6 to 68.2)
	2008	**52.7** (50.4 to 55.1)	50.4 (45.2 to 55.6)	56.4 (51.1 to 61.8)	51.5 (45.6 to 57.5)	53.2 (48.3 to 58.1)	52.4 (46.9 to 58.0)
	Change from 1994	**-11.6** (-14.5 to -8.7)	-12.1 (-18.8 to -5.3)	-7.3 (-14.0 to -0.7)	-16.1 (-23.0 to -9.3)	-9.7 (-16.0 to -3.5)	-11.9 (-18.6 to -5.2)
	P-value	***(<0.000)***	*(<0.000)*	*(0.031)*	*(<0.000)*	*(0.002)*	*(<0.000)*
**Fruit and vegetable consumption (portions per day)**	2001	**3.4** (3.3 to 3.5)	3.7 (3.5 to 3.9)	3.6 (3.5 to 3.8)	3.5 (3.3 to 3.7)	3.1 (3.0 to 3.3)	2.9 (2.7 to 3.1)
	2008	**3.7** (3.6 to 3.7)	3.9 (3.7 to 4.1)	3.9 (3.7 to 4.1)	3.7 (3.5 to 3.9)	3.6 (3.5 to 3.8)	3.2 (3.0 to 3.3)
	Change from 2001	***0.3** (0.2 to 0.4)*	*0.2 (-0.1 to 0.4)*	*0.3 (0.0 to 0.5)*	*0.2 (-0.1 to 0.5)*	*0.5 (0.3 to 0.7)*	*0.2 (0.0 to 0.5)*
	P-value	***(<0.000)***	*(0.124)*	*(0.025)*	*(0.212)*	*(<0.000)*	*(0.072)*
**Fruit and vegetable consumption (≥ 5 portions per day) (%)**	2001	**23.7** (22.4 to 25.0)	26.6 (23.8 to 29.5)	26.4 (23.6 to 29.3)	25.2 (22.2 to 28.1)	21.1 (18.6 to 23.6)	18.6 (15.6 to 21.5)
	2008	**27.8** (26.4 to 29.2)	29.6 (26.6 to 32.6)	31.0 (27.8 to 34.2)	29.5 (26.2 to 32.8)	27.5 (24.5 to 30.5)	21.0 (18.3 to 23.8)
	Change from 2001	**4.1** (2.2 to 6.0)	3.0 (-1.2 to 7.1)	4.6 (0.3 to 8.9)	4.3 (-0.1 to 8.7)	6.4 (2.5 to 10.3)	2.5 (-1.5 to 6.5)
	P-value	***(<0.000)***	*(0.162)*	*(0.037)*	*(0.053)*	*(0.001)*	*(0.228)*
