# Supplementary material for: Persistent socioeconomic inequalities in cardiovascular risk factors in England over 1994-2008: A time-trend analysis of repeated cross-sectional data
Source: BMC Public Health. 2012 Feb 14;12:129. doi: 10.1186/1471-2458-12-129 (PMC3342910; doi:10.1186/1471-2458-12-129)
Supplement: Additional file 9 — Absolute change in cardiovascular risk factors in women aged ≥ 55 years, by deprivation quintiles. The table shows the absolute change in cardiovascular risk factors between the most affluent and most deprived fifths over 1994-2008 with accompanying 95% confidence intervals. [file 1471-2458-12-129-S9.PDF]

ADDITIONAL FILE 9 Absolute change in cardiovascular risk factors in women aged ≥ 55 years, by deprivation quintiles (1/2)

		England	Q1<br>Most affluent	Q2	Q3	Q4	Q5<br>Most deprived
**Cigarette Smoking (%)**	1994	**20.5** (18.8 to 22.1)	11.9 (9.1 to 14.7)	15.6 (12.7 to 18.6)	18.9 (15.8 to 21.9)	23.8 (19.8 to 27.7)	32.6 (28.1 to 37.0)
	2008	**14.4** (13.0 to 15.8)	8.2 (6.3 to 10.1)	10.7 (8.4 to 13.0)	11.5 (8.9 to 14.0)	18.0 (14.8 to 21.2)	24.5 (20.4 to 28.7)
	Change from 1994	***-6.1*** *(-8.2 to -3.9)*	*-3.7 (-7.1 to -0.3)*	*-4.9 (-8.7 to -1.1)*	*-7.4 (-11.4 to -3.4)*	*-5.7 (-10.8 to -0.7)*	*-8.0 (-14.1 to -2.0)*
	P-value	*(<0.000)*	*(0.031)*	*(0.011)*	*(<0.000)*	*(0.026)*	*(0.009)*
**BMI (kg/m^2)**	1994	**27.1** (26.9 to 27.3)	26.3 (25.9 to 26.7)	27.3 (26.9 to 27.8)	27.4 (27.0 to 27.8)	27.4 (26.9 to 27.9)	27.2 (26.7 to 27.7)
	2008	**28.1** (27.9 to 28.3)	27.7 (27.3 to 28.1)	27.3 (26.9 to 27.8)	28.4 (27.9 to 28.8)	28.4 (27.9 to 28.9)	28.7 (28.1 to 29.3)
	Change from 1994	***1.0*** *(0.7 to 1.3)*	*1.4 (0.9 to 1.9)*	*0.0 (-0.6 to 0.6)*	*1.0 (0.3 to 1.6)*	*1.0 (0.3 to 1.7)*	*1.5 (0.7 to 2.3)*
	P-value	*(<0.000)*	*(<0.000)*	*(0.929)*	*(0.004)*	*(0.005)*	*(<0.000)*
**Obesity (%)**	1994	**23.9** (22.2 to 25.6)	17.8 (14.7 to 21.0)	26.1 (22.3 to 30.0)	24.2 (20.5 to 27.9)	26.1 (22.1 to 30.1)	25.1 (20.8 to 29.4)
	2008	**31.4** (29.6 to 33.3)	28.1 (24.8 to 31.3)	24.1 (20.5 to 27.6)	31.8 (27.6 to 35.9)	34.6 (30.4 to 38.9)	39.4 (34.4 to 44.5)
	Change from 1994	***7.6*** *(5.1 to 10.1)*	*10.2 (5.7 to 14.8)*	*-2.1 (-7.3 to 3.1)*	*7.6 (2.0 to 13.1)*	*8.5 (2.7 to 14.3)*	*14.3 (7.7 to 21.0)*
	P-value	*(<0.000)*	*(<0.000)*	*(0.436)*	*(0.008)*	*(0.004)*	*(<0.000)*
**Diabetes (%)**	1994	**3.8** (3.1 to 4.5)	3.1 (1.7 to 4.6)	3.3 (1.9 to 4.8)	2.8 (1.5 to 4.0)	4.7 (2.9 to 6.5)	5.1 (3.2 to 7.0)
	2006	**8.7** (7.4 to 9.9)	6.5 (4.2 to 8.7)	5.8 (3.7 to 7.9)	7.1 (4.8 to 9.4)	11.3 (8.0 to 14.7)	13.7 (9.6 to 17.9)
	Change from 1994	***4.9*** *(3.5 to 6.3)*	*3.4 (0.7 to 6.0)*	*2.4 (-0.1 to 5.0)*	*4.3 (1.8 to 6.9)*	*6.6 (2.8 to 10.4)*	*8.6 (4.0 to 13.2)*
	P-value	*(<0.000)*	*(0.014)*	*(0.060)*	*(0.001)*	*(0.001)*	*(<0.000)*
**High physical activity (%)**	1998	**11.9** (10.6 to 13.2)	13.0 (10.2 to 15.9)	12.0 (9.3 to 14.7)	11.0 (8.1 to 13.8)	11.9 (8.9 to 14.9)	11.4 (8.5 to 14.3)
	2008	**18.4** (17.0 to 19.9)	21.5 (18.5 to 24.5)	19.8 (16.7 to 22.9)	21.2 (17.8 to 24.6)	17.7 (14.5 to 20.9)	11.5 (8.4 to 14.5)
	Change from 1998	***6.6*** *(4.6 to 8.5)*	*8.5 (4.3 to 12.6)*	*7.8 (3.7 to 11.9)*	*10.2 (5.8 to 14.6)*	*5.8 (1.4 to 10.2)*	*0.1 (-4.1 to 4.3)*
	P-value	*(<0.000)*	*(<0.000)*	*(<0.000)*	*(<0.000)*	*(0.010)*	*(0.980)*
**SBP (mmHg)**	1994	**144.9** (144.0 to 145.8)	143.6 (141.6 to 145.7)	145.9 (143.9 to 147.9)	145.3 (143.5 to 147.1)	145.2 (143.2 to 147.1)	144.6 (142.6 to 146.6)
	2008	**134.3** (133.4 to 135.2)	133.6 (132.0 to 135.1)	133.6 (131.9 to 135.3)	132.4 (130.6 to 134.2)	134.4 (132.4 to 136.4)	138.1 (135.7 to 140.6)
	Change from 1994	***-10.6*** *(-11.9 to -9.4)*	*-10.0 (-12.6 to -7.5)*	*-12.3 (-14.9 to -9.7)*	*-12.9 (-15.5 to -10.4)*	*-10.8 (-13.6 to -7.9)*	*-6.5 (-9.6 to -3.3)*
	P-value	*(<0.000)*	*(<0.000)*	*(<0.000)*	*(<0.000)*	*(<0.000)*	*(<0.000)*
**SBP ≥ 140 mmHg (%)**	1994	**54.2** (52.0 to 56.4)	51.8 (47.1 to 56.6)	53.2 (48.3 to 58.1)	55.2 (50.8 to 59.6)	56.1 (51.0 to 61.2)	54.6 (49.7 to 59.6)
	2008	**34.6** (32.4 to 36.8)	32.4 (28.5 to 36.4)	34.4 (30.3 to 38.5)	31.4 (26.8 to 36.0)	33.3 (28.2 to 38.5)	42.6 (36.2 to 48.9)
	Change from 1994	**-19.6** (-22.7 to -16.5)	-19.4 (-25.6 to -13.3)	-18.8 (-25.2 to -12.4)	-23.8 (-30.2 to -17.5)	-22.8 (-30.0 to -15.5)	-12.1 (-20.1 to -4.0)
	P-value	*(<0.000)*	*(<0.000)*	*(<0.000)*	*(<0.000)*	*(<0.000)*	*(0.003)*

ADDITIONAL FILE 9 Absolute change in cardiovascular risk factors in women aged ≥ 55 years, by deprivation quintiles (2/2)

		**England**	**Q1 Most affluent**	**Q2**	**Q3**	**Q4**	**Q5 Most deprived**
**Total cholesterol (mmol/l)**	1994	**6.9** (6.8 to 6.9)	6.8 (6.7 to 6.9)	6.8 (6.7 to 7.0)	6.9 (6.8 to 7.0)	6.9 (6.7 to 7.0)	6.9 (6.8 to 7.0)
	2008	**5.9** (5.8 to 5.9)	5.9 (5.8 to 6.0)	5.9 (5.8 to 6.1)	5.9 (5.7 to 6.0)	5.8 (5.7 to 6.0)	5.7 (5.6 to 5.9)
	Change from 1994	***-1.0*** *(-1.1 to -0.9)*	*-0.9 (-1.0 to -0.7)*	*-0.9 (-1.1 to -0.7)*	*-1.0 (-1.2 to -0.9)*	*-1.0 (-1.2 to -0.8)*	*-1.2 (-1.4 to -0.9)*
	P-value	***(<0.000)***	*(<0.000)*	*(<0.000)*	*(<0.000)*	*(<0.000)*	*(<0.000)*
**Total cholesterol ≥ 5.0 mmol/l (%)**	1994	**95.0** (94.0 to 95.9)	95.2 (93.1 to 97.3)	95.6 (93.4 to 97.7)	96.5 (94.8 to 98.1)	93.5 (90.7 to 96.3)	93.7 (91.3 to 96.1)
	2008	**76.9** (74.8 to 79.1)	83.4 (79.7 to 87.1)	78.5 (74.0 to 83.0)	77.4 (73.0 to 81.9)	72.6 (67.4 to 77.9)	71.3 (65.0 to 77.5)
	Change from 1994	**-18.0** (-20.4 to -15.7)	-11.8 (-16.0 to -7.5)	-17.1 (-22.1 to -12.1)	-19.0 (-23.8 to -14.3)	-20.9 (-26.8 to -14.9)	-22.4 (-29.1 to -15.7)
	P-value	***(<0.000)***	*(<0.000)*	*(<0.000)*	*(<0.000)*	*(<0.000)*	*(<0.000)*
**Fruit and vegetable consumption (portions per day)**	2001	**3.7** (3.6 to 3.8)	4.3 (4.0 to 4.5)	4.0 (3.8 to 4.2)	3.7 (3.5 to 3.9)	3.4 (3.2 to 3.6)	2.9 (2.7 to 3.1)
	2008	**4.0** (3.9 to 4.1)	4.3 (4.2 to 4.5)	4.3 (4.1 to 4.4)	4.1 (3.9 to 4.3)	4.0 (3.7 to 4.2)	3.2 (3.0 to 3.4)
	Change from 2001	***0.3*** *(0.2 to 0.5)*	*0.1 (-0.2 to 0.4)*	*0.3 (0.0 to 0.6)*	*0.4 (0.1 to 0.7)*	*0.5 (0.2 to 0.8)*	*0.3 (0.0 to 0.6)*
	P-value	***(<0.000)***	*(0.592)*	*(0.032)*	*(0.014)*	*(0.001)*	*(0.050)*
**Fruit and vegetable consumption (≥ 5 portions per day) (%)**	2001	**26.0** (24.3 to 27.7)	35.2 (31.4 to 39.1)	27.5 (24.1 to 30.8)	26.0 (22.2 to 29.8)	22.4 (18.8 to 25.9)	17.9 (14.3 to 21.6)
	2008	**31.1** (29.4 to 32.8)	36.3 (32.6 to 39.9)	34.7 (31.4 to 38.0)	33.6 (29.8 to 37.4)	31.2 (27.3 to 35.1)	18.9 (15.2 to 22.5)
	Change from 2001	**5.1** (2.7 to 7.5)	1.0 (-4.3 to 6.3)	7.2 (2.5 to 11.9)	7.6 (2.2 to 13.0)	8.8 (3.5 to 14.1)	0.9 (-4.3 to 6.1)
	P-value	***(<0.000)***	*(0.700)*	*(0.003)*	*(0.006)*	*(0.001)*	*(0.726)*
