# Supplementary material for: Persistent socioeconomic inequalities in cardiovascular risk factors in England over 1994-2008: A time-trend analysis of repeated cross-sectional data
Source: BMC Public Health. 2012 Feb 14;12:129. doi: 10.1186/1471-2458-12-129 (PMC3342910; doi:10.1186/1471-2458-12-129)
Supplement: Additional file 10 — Annual change in cardiovascular risk factors in men, by deprivation quintile and age. The table shows the annual change in cardiovascular risk factors for all deprivation fifths with accompanying 95% confidence intervals. [file 1471-2458-12-129-S10.PDF]

Additional file 10: Annual change in cardiovascular risk factors in men, by deprivation quintile and age (1/2)[§]

	16-54						≥ 55 years					
**Trend[¶]**	**England**	**Q1 (affluent)**	**Q2**	**Q3**	**Q4**	**Q5 (deprived)**	**England**	**Q1 (affluent)**	**Q2**	**Q3**	**Q4**	**Q5 (deprived)**
Current smoking												
**Year**	**0.986**	**0.984**	**0.984**	**0.986**	**0.987**	**0.988**	**0.980**	**0.970**	**0.973**	**0.974**	**0.988**	**0.987**
95% CI	(0.983,0.989)	(0.975,0.992)	(0.976,0.992)	(0.979,0.993)	(0.981,0.994)	(0.982,0.993)	(0.975,0.986)	(0.954,0.985)	(0.959,0.988)	(0.962,0.986)	(0.977,0.999)	(0.977,0.996)
P-value	(<0.000)	(<0.000)	(<0.000)	(<0.000)	(<0.000)	(<0.000)	(<0.000)	(<0.000)	(<0.000)	(<0.000)	(0.035)	(0.007)
**Year^2**												
95% CI	-	-	-	-	-	-	-	-	-	-	-	-
P-value	(0.011)	(0.668)	(0.511)	(0.019)	(0.245)	(0.078)	(0.296)	(0.342)	(0.703)	(0.158)	(0.228)	(0.952)
BMI (kg/m^2)												
**Year**	**0.58**	**0.24**	**0.35**	**0.27**	**0.27**	**0.26**	**0.36**	**0.27**	**0.37**	**0.38**	**0.38**	**0.38**
95% CI	(0.44,0.72)	(0.17,0.31)	(0.27,0.42)	(0.19,0.34)	(0.19,0.36)	(0.18,0.35)	(0.31,0.40)	(0.19,0.35)	(0.29,0.45)	(0.30,0.47)	(0.28,0.48)	(0.27,0.50)
P-value	(<0.000)	(<0.000)	(<0.000)	(<0.000)	(<0.000)	(<0.000)	(<0.000)	(<0.000)	(<0.000)	(<0.000)	(<0.000)	(<0.000)
**Year^2**	**-0.02**											
95% CI	(-0.03,-0.01)	-	-	-	-	-	-	-	-	-	-	-
P-value	(<0.000)	(0.044)	(0.238)	(0.015)	(0.011)	(0.312)	(0.595)	(0.751)	(0.664)	(0.393)	(0.505)	(0.737)
Obesity (% BMI ≥30)												
**Year**	**1.092**	**1.039**	**1.048**	**1.097**	**1.036**	**1.032**	**1.039**	**1.035**	**1.037**	**1.044**	**1.040**	**1.040**
95% CI	(1.071,1.114)	(1.029,1.050)	(1.038,1.059)	(1.052,1.145)	(1.026,1.046)	(1.022,1.042)	(1.034,1.044)	(1.023,1.047)	(1.027,1.048)	(1.033,1.054)	(1.029,1.051)	(1.028,1.052)
P-value	(<0.000)	(<0.000)	(<0.000)	(<0.000)	(<0.000)	(<0.000)	(<0.000)	(<0.000)	(<0.000)	(<0.000)	(<0.000)	(<0.000)
**Year^2**	**0.997**			**0.996**								
95% CI	(0.996,0.998)	-	-	(0.994,0.999)	-	-	-	-	-	-	-	-
P-value	(<0.000)	(0.014)	(0.019)	(0.006)	(0.040)	(0.040)	(0.374)	(0.784)	(0.790)	(0.218)	(0.580)	(0.756)
Diabetes[¶]												
**Year**	**1.062**	**1.014**	**1.054**	**1.077**	**1.080**	**1.072**	**1.059**	**1.037**	**1.068**	**1.066**	**1.049**	**1.071**
95% CI	(1.033,1.091)	(0.950,1.082)	(0.987,1.125)	(1.015,1.143)	(1.017,1.147)	(1.020,1.128)	(1.041,1.076)	(1.001,1.074)	(1.027,1.110)	(1.030,1.102)	(1.009,1.089)	(1.003,1.110)
P-value	(<0.000)	(0.673)	(0.119)	(0.015)	(0.012)	(0.007)	(<0.000)	(0.043)	(0.001)	(<0.000)	(0.015)	(<0.000)
High physical activity[¶]												
**Year**	**1.027**	**1.034**	**1.023**	**1.022**	**1.035**	**1.021**	**1.033**	**1.038**	**1.030**	**1.052**	**1.019**	**1.028**
95% CI	(1.020,1.034)	(1.018,1.051)	(1.008,1.039)	(1.007,1.037)	(1.020,1.050)	(1.004,1.038)	(1.020,1.047)	(1.011,1.065)	(1.002,1.059)	(1.024,1.081)	(0.990,1.048)	(0.990,1.067)
P-value	(<0.000)	(<0.000)	(0.003)	(0.004)	(0.000)	(0.016)	(<0.000)	(0.006)	(0.036)	(<0.000)	(0.209)	(0.151)
SBP (mmHg)												
**Year**	**-0.19**	**-0.24**	**-0.15**	**-0.16**	**-0.19**	**-0.22**	**-0.43**	**-0.48**	**-0.40**	**-0.40**	**-0.39**	**-0.49**
95% CI	(-0.22,-0.17)	(-0.29,-0.19)	(-0.21,-0.10)	(-0.22,-0.11)	(-0.24,-0.13)	(-0.28,-0.16)	(-0.47,-0.39)	(-0.56,-0.41)	(-0.48,-0.32)	(-0.48,-0.31)	(-0.48,-0.29)	(-0.59,-0.38)
P-value	(<0.000)	(<0.000)	(<0.000)	(<0.000)	(<0.000)	(<0.000)	(<0.000)	(<0.000)	(<0.000)	(<0.000)	(<0.000)	(<0.000)
**Year^2**												
95% CI	-	-	-	-	-	-	-	-	-	-	-	-
P-value	(0.997)	(0.226)	(0.939)	(0.128)	(0.916)	(0.716)	(0.108)	(0.345)	(0.349)	(0.946)	(0.242)	(0.415)

Additional file 10: Annual change in cardiovascular risk factors in men, by deprivation quintile and age (2/2) §

	16-54						≥ 55 years					
**Trend ‖**	**England**	**Q1 (affluent)**	**Q2**	**Q3**	**Q4**	**Q5 (deprived)**	**England**	**Q1 (affluent)**	**Q2**	**Q3**	**Q4**	**Q5 (deprived)**
High blood pressure (% SBP ≥ 140 mmHg)												
**Year**	**0.980**	**0.972**	**0.989**	**0.985**	**0.983**	**0.971**	**0.974**	**0.974**	**0.975**	**0.975**	**0.975**	**0.972**
95% CI	(0.974,0.986)	(0.960,0.984)	(0.976,1.002)	(0.972,0.998)	(0.970,0.995)	(0.959,0.984)	(0.971,0.977)	(0.968,0.981)	(0.968,0.982)	(0.968,0.982)	(0.968,0.982)	(0.964,0.979)
P-value	(<0.000)	(<0.000)	(0.095)	(0.022)	(0.008)	(<0.000)	(<0.000)	(<0.000)	(<0.000)	(<0.000)	(<0.000)	(<0.000)
**Year^2**												
95% CI	-	-	-	-	-	-	-	-	-	-	-	-
P-value	(0.061)	(0.518)	(0.051)	(0.040)	(0.844)	(0.382)	(0.016)	(0.288)	(0.876)	(0.111)	(0.081)	(0.154)
Total cholesterol (mmol/l)¶												
**Year**	**-0.4**	**-0.4**	**-0.3**	**-0.4**	**-0.3**	**-0.4**	**-1.1**	**-1.1**	**-1.0**	**-1.0**	**-1.1**	**-1.3**
95% CI	(-0.5,-0.3)	(-0.5,-0.2)	(-0.5,-0.2)	(-0.6,-0.3)	(-0.4,-0.1)	(-0.6,-0.3)	(-1.2,-1.0)	(-1.3,-0.9)	(-1.2,-0.8)	(-1.2,-0.8)	(-1.5,-0.8)	(-1.6,-1.1)
P-value	(<0.000)	(<0.000)	(<0.000)	(<0.000)	(0.001)	(<0.000)	(<0.000)	(<0.000)	(<0.000)	(<0.000)	(<0.000)	(<0.000)
Raised cholesterol (% TC ≥ 5.0 mmol/l)¶												
**Year**	**0.990**	**0.987**	**0.993**	**0.989**	**0.990**	**0.988**	**0.976**	**0.979**	**0.981**	**0.978**	**0.971**	**0.968**
95% CI	(0.988,0.992)	(0.981,0.992)	(0.988,0.998)	(0.985,0.995)	(0.985,0.995)	(0.982,0.993)	(0.973,0.978)	(0.974,0.984)	(0.976,0.986)	(0.972,0.983)	(0.964,0.977)	(0.961,0.975)
P-value	(<0.000)	(<0.000)	(0.006)	(<0.000)	(<0.000)	(<0.000)	(<0.000)	(<0.000)	(<0.000)	(<0.000)	(<0.000)	(<0.000)
Fruit and vegetable consumption (portions)												
**Year**	**6.3**	**3.1**	**6.4**	**5.2**	**10.2**	**7.8**	**2.7**	**1.4**	**2.2**	**5.1**	**2.8**	**3.1**
95% CI	(4.2,8.5)	(-1.0,7.3)	(2.4,10.5)	(0.5,9.9)	(5.5,14.9)	(2.2,13.3)	(0.7,4.7)	(-1.7,4.5)	(-0.9,5.2)	(0.9,9.3)	(-1.6,7.3)	(-3.3,9.6)
P-value	(<0.000)	(0.138)	(0.002)	(0.029)	(<0.000)	(0.006)	(0.007)	(0.381)	(0.167)	(0.016)	(0.214)	(0.337)
**Year^2**												
95% CI	-	-	-	-	-	-	-	-	-	-	-	-
P-value	(0.447)	(0.501)	(0.494)	(0.846)	(0.765)	(0.081)	(0.231)	(0.944)	(0.507)	(0.873)	(0.035)	(0.714)
Fruit and vegetable consumption (% ≥ 5 portions per day)												
**Year**	**1.038**	**1.032**	**1.040**	**1.036**	**1.046**	**1.034**	**1.025**	**1.020**	**1.005**	**1.003**	**1.030**	**1.061**
95% CI	(1.027,1.048)	(1.011,1.053)	(1.017,1.064)	(1.012,1.061)	(1.023,1.070)	(1.005,1.063)	(1.013,1.037)	(1.000,1.040)	(0.984,1.025)	(1.008,1.059)	(1.001,1.059)	(1.024,1.099)
P-value	(<0.000)	(0.002)	(0.001)	(0.003)	(<0.000)	(0.020)	(<0.000)	(0.050)	(0.651)	(0.010)	(0.042)	(0.001)
**Year^2**												
95% CI	-	-	-	-	-	-	-	-	-	-	-	-
P-value	(0.030)	(0.153)	(0.641)	(0.504)	(0.013)	(0.797)	(0.401)	(0.129)	(0.805)	(0.801)	(0.631)	(0.677)

§Values are prevalence ratios (PR) for annual change (smoking, obesity, diabetes, high physical activity, high blood pressure, raised cholesterol, and consuming five or more portions of fruit and vegetables per day) or level (body mass index, systolic blood pressure, total cholesterol, and fruit and vegetable consumption). Log-binomial and log-linear regression used for binary and continuous risk factors respectively. Models fitted separately to each deprivation quintile (adjusted for age). Negative numbers/PR below 1 indicate a decreasing trend, positive numbers/PR above 1 an increasing trend. [Tests of socioeconomic differentials in the pace of change are shown for binary risk factors in Tables 2 and 3. The p-values for the interaction terms assess a) whether the change in risk factor levels/PR over 1994-2008 differed according to IMD; and equivalently b) whether the differences in risk factor levels/PR by IMD changed over time].

‖A quadratic trend (year^2) indicates a significant but nonlinear trend in the data over time. A linear trend (year) is depicted with a straight line; a quadratic trend as a curve with one bend. Trends that include significant quadratic and linear components demonstrate nonlinear change in addition to an overall increase

or decrease over time (and so are not comparable to models just containing a linear term). Two models were fitted to each deprivation quintile. Model 1 contained just the linear trend; Model 2 included linear and quadratic trends (year + year^2). Estimates from Model 2 are shown if the quadratic term was significant at the 1% level. Quadratic terms not significant at the 1% level were removed from the model leaving just the linear trend. For quadratic terms not significant at the 1% level we show the linear trend from Model 1 - but show the p-value for the non-significant quadratic term obtained from Model 2.

[¶] Only linear trends fitted due to limited data points

CI confidence interval; SBP systolic blood pressure; TC total cholesterol; BMI body mass index
